# Characterization of codon usage pattern in SARS-CoV-2

**DOI:** 10.1186/s12985-020-01395-x

**Published:** 2020-09-14

**Authors:** Wei Hou

**Affiliations:** Tianjin Second People’s Hospital and Tianjin Institute of Hepatology, 7 Sudi South Road, Nankai District, Tianjin, 300192 China

**Keywords:** COVID-19, Coronaviruses, SARS-CoV-2, Codon usage pattern

## Abstract

The outbreak of coronavirus disease 2019 (COVID-19) due to severe acute respiratory syndrome coronavirus 2 (SARS-CoV-2) has posed significant threats to international health. The genetic traits as well as evolutionary processes in this novel coronavirus are not fully characterized, and their roles in viral pathogenesis are yet largely unknown. To get a better picture of the codon architecture of this newly emerging coronavirus, in this study we perform bioinformatic analysis, based on publicly available nucleotide sequences of SARS-CoV-2 along with those of other members of human coronaviruses as well as non-human coronaviruses in different hosts, to take a snapshot of the genome-wide codon usage pattern of SARS-CoV-2 and uncover that all over-represented codons end with A/U and this newly emerging coronavirus has a relatively low codon usage bias, which is shaped by both mutation pressure and natural selection. Additionally, there is slight variation in the codon usage pattern among the SARS-CoV-2 isolates from different geo-locations. Furthermore, the overall codon usage pattern of SARS-CoV-2 is generally similar to that of its phylogenetic relatives among non-human betacoronaviruses such as RaTG13. Taken together, we comprehensively analyze the characteristics of codon usage pattern in SARS-CoV-2 via bioinformatic approaches. The information from this research may not only be helpful to get new insights into the evolution of SARS-CoV-2, but also have potential value for developing coronavirus vaccines.

## Introduction

Coronaviruses (CoVs) belong to the family *Coronavirdiae* comprises large, single, positive-sense single-stranded RNA viruses including four genera of CoVs, namely, *Alphacoronavirus*, *Betacoronavirus*, *Deltacoronavirus*, and *Gammacoronavirus* [1]. Several coronavirus species were extensively known to cause human disease [1–3], including two alphacoronaviruses (HCoV-229E, HCoV-NL63) and four betacoronaviruses (HCoV-OC43, HCoV-HKU, sereve acute respiratory syndrome coronavirus SARS-CoV and Middle East respiratory syndrome coronavirus MERS-CoV). Very recently, the outbreak of coronavirus disease 2019 (COVID-19) due to severe acute respiratory syndrome coronavirus 2 (SARS-CoV-2) has posed significant threats to international health [4–9]. However, the genetic traits as well as evolutionary processes in this newly emerging coronavirus are not fully characterized, and their roles in viral pathogenesis are yet largely unknown. To further explore the codon usage pattern of SARS-CoV-2 to get a better picture of the codon architecture of this novel coronavirus, genomic sequences of the SARS-CoV-2 and other representative coronaviruses were analyzed via bioinformatic approaches.

## Materials and methods

### Genomic sequences acquisition

Genomic sequences of the SARS-CoV-2 Wuhan-Hu-1 (MN908947.3), other representative coronaviruses including human coronaviruses such as HCoV-229E (AF304460.1), HCoV-NL63 (AY567487.2), HCoV-OC43(AY585228.1), HCoV-HKU1(MH940245.1), and SARS-CoV (strain: Urbani, AY278741.1; strain: Tor2, AY274119.3), MERS-CoV (strain: HCoV-EMC, JX869059.2) and nonhuman coronaviruses (Supplementary Table 1) were all retrieved from GenBank.

### Phylogenetic analysis

Phylogenetic tree of the whole genome sequences of coronaviruses were constructed by using MEGA software version 6.0 (http://www.megasoftware.net) with the maximum likelihood algorithm and Kimura 2-parameter model with 1000 bootstrap replicates.

### Codon usage pattern analysis

The basic nucleotide composition (A%, U%, C%, and G%), AU and GC contents, relative synonymous codon usage (RSCU) were analyzed using MEGA software. The parameters of codon usage bias including intrinsic codon bias index (ICDI), codon bias index (CBI), effective number of codons (ENC) were analyzed using CAIcal [10] and COUSIN programs (http://cousin.ird.fr/index.php).

### Cluster analysis

Cluster analysis (Heat map) was performed using CIMminer (https://discover.nci.nih.gov/cimminer/).

## Results and discussion

**Fig. 1:**
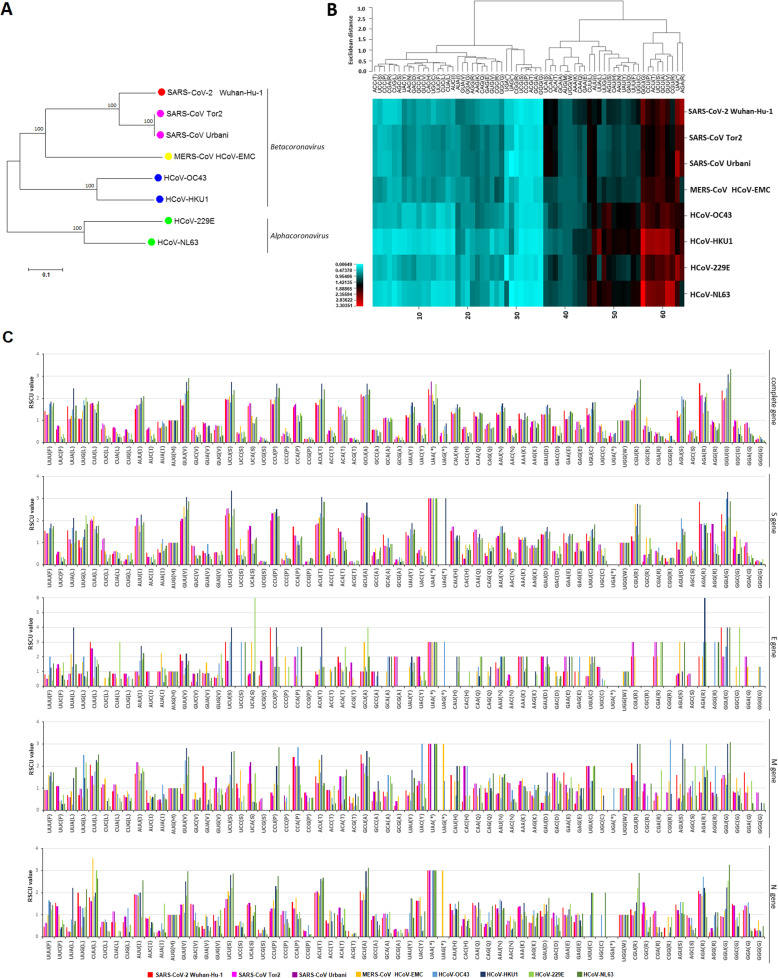
Bioinformatic analyses of SARS-CoV-2 and other human coronaviruses. **a** Maximum likelihood phylogenetic tree of the whole genome sequences of SARS-CoV-2 Wuhan-Hu-1 (MN908947.3) and related coronaviruses including human coronavirus HCoV-229E (AF304460.1), HCoV-NL63 (AY567487.2), HCoV-OC43(AY585228.1), HCoV-HKU1(MH940245.1), and SARS-CoV (strain: Urbani, AY278741.1; strain: Tor2, AY274119.3), MERS-CoV (strain: HCoV-EMC, JX869059.2). **b** Heat map of RSCU values for the complete coding sequences of SARS-CoV-2 and other human coronaviruses. The heatmap analysis was performed using CIMminer. Each column represents a codon. Codons with higher RSCU values are highlighted with a red background. **c** The profiles of the relative synonymous codon usage for different genes of SARS-CoV-2 and other human coronaviruses. RSCU values were shown as the vertical bar graph. S:spike; E:envelop; M: membrane; N: nucleocapsid

Phylogenetic analysis of human coronavirus genomes (Fig. 1a) revealed that the newly identified coronavirus SARS-CoV-2 Wuhan-Hu-1 sequence was closer to SARS-CoV Tor2 as well as SARS-CoV Urbani, and relatively distant to two alphacoronaviruses (HCoV-229E, HCoV-NL63).

Nucleotide composition analysis (Supplementary Figure 1A) revealed that SARS-CoV-2 Wuhan-Hu-1 had the highest compositional value of U% (32.2) which was followed by A% (29.9), and similar composition of G% (19.6) and C% (18.3). At the third position, we observed that nucleotide U also occurred most frequently. Thus, most codons of SARS-CoV-2 Wuhan-Hu-1 tended to be U ending. Moreover, the mean GC and AU compositions (Supplementary Figure 1B) were 37.9 and 62.1% (SARS-CoV-2 Wuhan-Hu-1), 41.0 and 59.0% (SARS-CoV Tor2), 40.8 and 59.2% (SARS-CoV Urbani), 41.5 and 58.5% (MERS-CoV HCoV-EMC), 36.8 and 63.2% (HCoV-OC43), 32.0 and 68.0% (HCoV-HKU1), 38.0 and 62.0% (HCoV-229E), 34.4 and 65.6% (HCoV-NL63), respectively indicating that SARS-CoV-2 Wuhan-Hu-1 as well as other human coronaviruses in this study were all AU rich, which was consistent with recent reports [11–15].

RSCU analysis of the complete coding sequences of SARS-CoV-2 Wuhan-Hu-1 revealed that all the over-represented codons (RSCU value > 1.6) ended with A/U whereas most of the under-represented codons (RSCU value < 0.6) ended with C/G (Supplementary Table 2). The highest RSCU value for the codon was AGA for R (2.67) amino acid and the lowest was UCG for S (0.11). The heatmap analysis (Fig. 1b) further revealed that all human coronaviruses analyzed in this study share the over-represented codons (UAA, GGU, GCU, UCU, GUU, CCU, ACU) and the average RSCU value > 2.0, whereas UCA were over-represented only in SARS-CoV-2 and SARS-CoV.

**Fig. 2:**
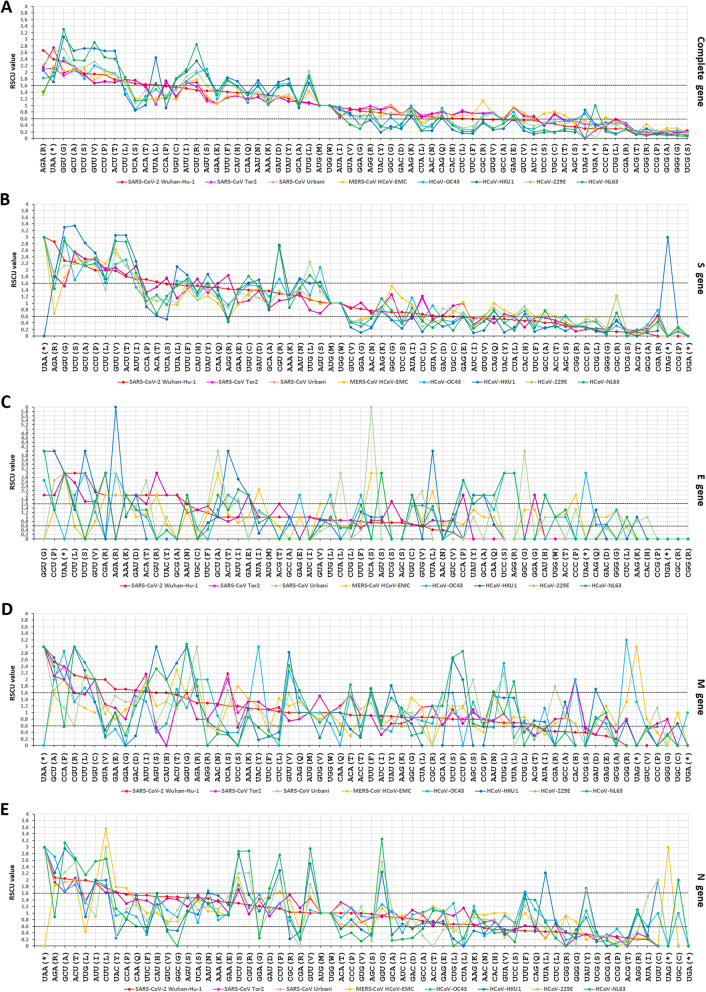
Over-represented codons (RSCU value > 1.6) and under-represented codons (RSCU value < 0.6) were compared among SARS-CoV-2 and other human coronaviruses. **a** Complete gene; **b** S gene; **c** E gene; **d** M gene; **e** N gene

The profiles of codon usage patterns among different genes of human coronaviruses were further analyzed (Figs. 1c and 2). As for spike (S) gene, all human coronaviruses analyzed in this study shared the over-represented codons (UCU, GCU, CUU, GUU, ACU) and all ended with U, whereas two codons (CCA, ACA) were over-represented only in SARS-CoV-2. In addition, SARS-CoV-2 did not use CGA for arginine nor CCG for proline. As for envelop (E) gene, two codons (UAC, GCG) were over-represented only in SARS-CoV-2 and SARS-CoV. All human coronaviruses analyzed in this study did not use two synonymous codons (CGC, CGG) for arginine as well as CCG for proline and UGA for stop codon at all. SARS-CoV-2 and SARS-CoV did not use CAA for glutamine nor UAU for tyrosine, whereas they use GCG for alanine, AUC for isoleucine, UCG and AGC for serine. As for membrane (M) gene, three codons (GUA, GAA, GGA) were over-represented only in SARS-CoV-2. As for nucleocapsid (N) gene, all human coronaviruses analyzed in this study share the over-represented codons (GCU, ACU, CUU) and all ended with U. The average RSCU values of GCU in complete gene, S gene, E gene, M gene and N gene in all human coronaviruses analyzed in this study were 2.22, 2.12, 1.79, 2.13, 2.16, respectively. GCU for alanine was identified as the highly preferred codon among the human coronaviruses.

**Fig. 3:**
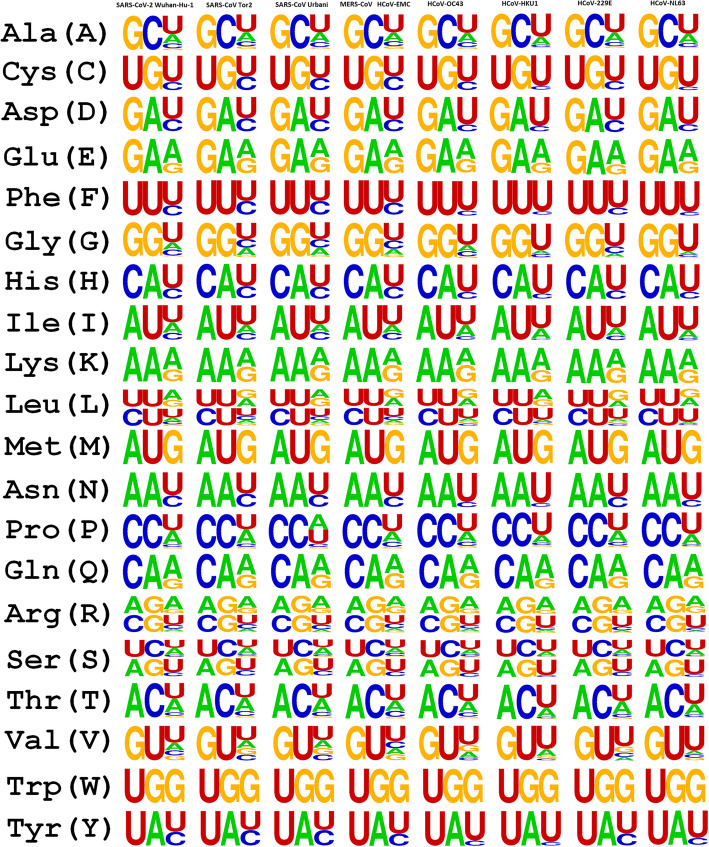
Graphical representation of synonymous codon usage pattern of each amino acid among SARS-CoV-2 and other human coronaviruses using Weblogo (https://weblogo.berkeley.edu/logo.cgi)

Amino acids are degenerate and each amino acid has different number of synonymous codons except for methionine (Met, M) and tryptophan (Trp, W). The overall amino acid usage of the human coronaviruses was shown in Supplementary Figure 2. Leucine and valine were the two most frequently used amino acids in all human coronaviruses analyzed in this study, CUU and GUU were preferred codons for leucine and valine, respectively (Fig. 3), whereas tryptophan, histidine and methionine were the three least used ones, which was consistent with recent report [14].

To further estimate the degree of codon usage bias, intrinsic codon bias index (ICDI), codon bias index (CBI) and effective number of codons (ENC) values were calculated (Table 1). ICDI value (0.144), CBI value (0.306) and ENC value (45.38) all exhibited relatively low codon usage bias of SARS-CoV-2, similar to SARS-CoV Tor2, SARS-CoV Urbani, MERS-CoV HCoV-EMC, HCoV-OC43, HCoV-229E whereas different from HCoV-HKU1 (ICDI 0.372; CBI 0.532; ENC 35.617) and HCoV-NL63 (ICDI 0.307; CBI 0.476; ENC 37.275), which exhibited moderate codon usage bias.

**Table 1:** The parameters of codon usage bias among the coronaviruses analyzed in this study

Coronaviruses	GenBank Accession	Genome Length (nt)	ICDI	CBI	ENC
SARS-CoV-2 Wuhan-Hu-1	MN908947.3	29,903	0.144	0.306	45.38
SARS-CoV Tor2	AY274119.3	29,751	0.075	0.223	49.746
SARS-CoV Urbani	AY278741.1	29,727	0.08	0.228	48.965
MERS-CoV HCoV-EMC	JX869059.2	30,119	0.082	0.248	50.033
HCoV-OC43	AY585228.1	30,741	0.213	0.367	43.794
HCoV-HKU1	MH940245.1	29,811	0.372	0.532	35.617
HCoV-229E	AF304460.1	27,317	0.172	0.358	43.45
HCoV-NL63	AY567487.2	27,553	0.307	0.476	37.275

We next attempted to determine the forces influencing the codon usage bias. Accumulating evidence suggests that the formation of codon usage bias is affected by many factors, and two generally accepted major forces are mutation pressure and natural selection [16]. Other influential factors include gene expression level, gene length, GC content, GC contents at the third base of one codon (GC3), RNA stability, hydrophilicity, and hydrophobicity, etc. When G or C is in high or low proportion at the third position of the codon, mutational pressure is involved [17]. From Supplementary Figure 1, it clearly showed that both G3 and C3 were lower than A3 and U3, suggesting the contribution of mutational force acting on codon usage pattern. Moreover, all preferred codons were A/U ending (Figs. 1b, c and 2), which further suggested that mutational force contributed to shape codon usage in this virus. Furthermore, to better understand the relation between gene composition and codon usage bias, an ENC–GC3 scatter diagram of ENC versus GC3S (ENC plotted against G + C content at the third codon position) was constructed. When codon usage pattern is only affected by GC3 resulting from mutation pressure, the expected ENC values should be just on the solid curved line. As shown in Supplementary Figure 3, all points lie together under the expected ENC curve, indicating that some independent factors, such as natural selection might also play a role in codon usage bias of human coronaviruses.

Apart from human, many animal species can also be infected by different types of coronaviruses. Previous studies have shown that some animals such as bats are believed to represent the original reservoir of several human-infecting coronaviruses [1]. In order to provide additional information to better understand the evolution of SARS-CoV-2, we further compared the codon usage pattern of SARS-CoV-2 and non-human coronaviruses (Supplementary Table 1).

**Fig. 4:**
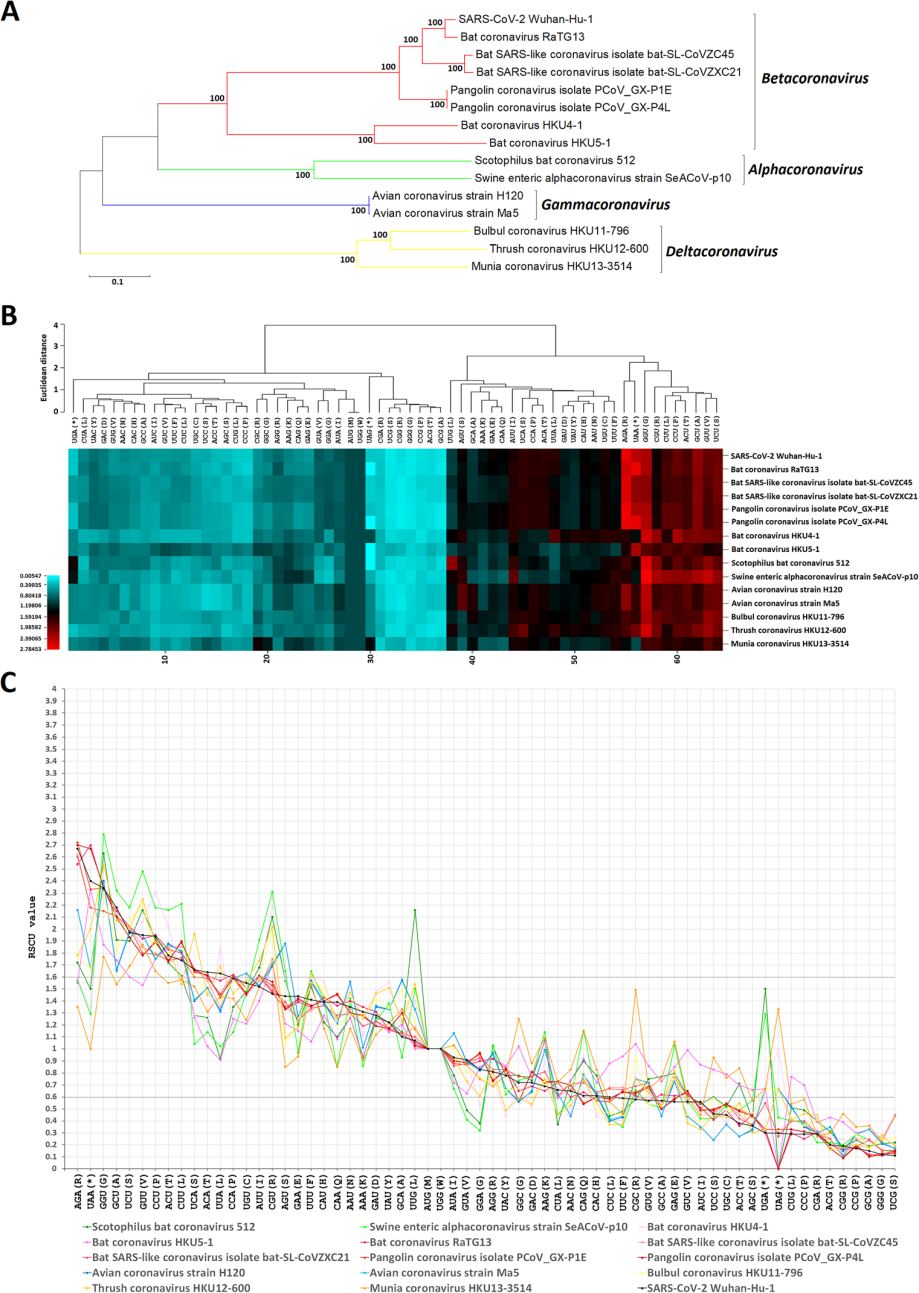
Comparative analysis of SARS-CoV-2 and non-human coronaviruses. **a** Maximum likelihood phylogenetic tree of the whole genome sequences of SARS-CoV-2 Wuhan-Hu-1 (MN908947.3) and non-human coronaviruses including Scotophilus bat coronavirus 512(NC_009657.1), Swine enteric alphacoronavirus strain SeACoV-p10(MK977618.1), Bat coronavirus HKU4–1(NC_009019.1), Bat coronavirus HKU5–1(NC_009020.1), Bat coronavirus RaTG13(MN996532.1), Bat SARS-like coronavirus isolate bat-SL-CoVZC45(MG772933.1), Bat SARS-like coronavirus isolate bat-SL-CoVZXC21(MG772934.1), Pangolin coronavirus isolate PCoV_GX-P1E(MT040334.1), Pangolin coronavirus isolate PCoV_GX-P4L(MT040333.1), Avian coronavirus strain H120(MK071267.1), Avian coronavirus strain Ma5(KY626045.1), Bulbul coronavirus HKU11–796(FJ376620.1), Thrush coronavirus HKU12–600(NC_011549.1), Munia coronavirus HKU13–3514(NC_011550.1). **b** Heat map of RSCU values for the complete coding sequences of SARS-CoV-2 and non-human coronaviruses. The heatmap analysis was performed using CIMminer. Each column represents a codon. Codons with higher RSCU values are highlighted with a red background. **c** The profiles of the relative synonymous codon usage for complete gene of SARS-CoV-2 and non-human coronaviruses. Over-represented codons (RSCU value > 1.6) and under-represented codons (RSCU value < 0.6) were shown as line graph

**Fig. 5:**
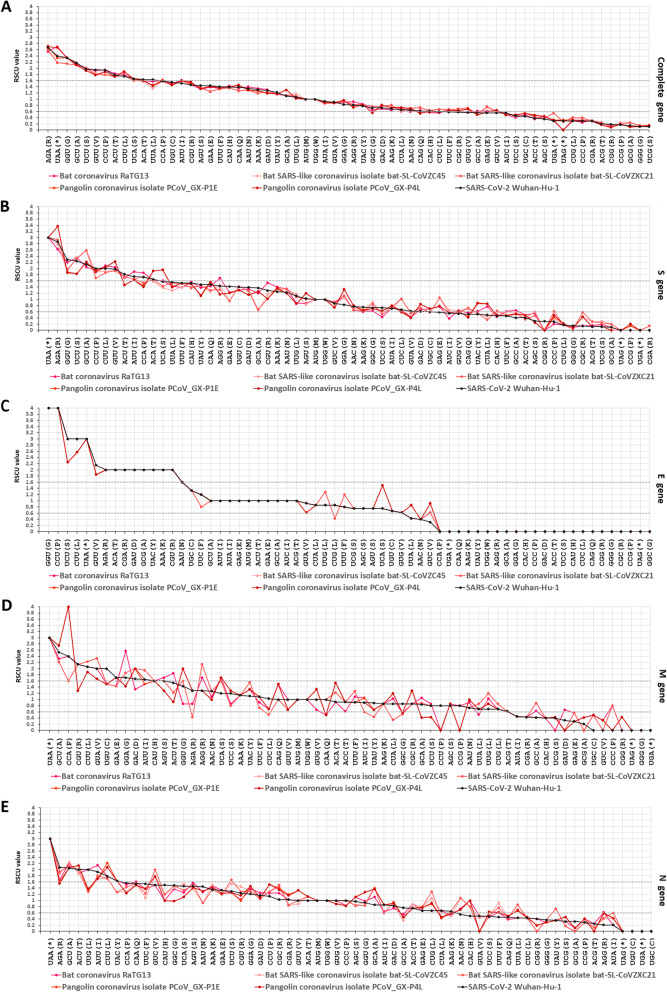
Over-represented codons (RSCU value > 1.6) and under-represented codons (RSCU value < 0.6) were compared among SARS-CoV-2 and its phylogenetic relatives including RaTG13, Bat-SL-CoVZC45, Bat-SL-CoVZXC21, PCoV_GX-P1E, PCoV_GX-P4L. **a** Complete gene; **b** S gene; **c** E gene; **d** M gene; **e** N gene

Phylogenetic analysis (Fig. 4a) showed that SARS-CoV-2 was most closely related to recently reported Bat coronavirus RaTG13 [8]. Nucleotide composition analysis (Supplementary Figure 4) revealed that similar to SARS-CoV-2 Wuhan-Hu-1, all the non-human coronaviruses analyzed in this study had the highest compositional value of U% and nucleotide U occurred most frequently at the third position. The heatmap analysis (Fig. 4b) revealed that SARS-CoV-2 and all the non-human coronaviruses analyzed in this study shared the over-represented codons (GGU, UCU, CCU) and all ended with U, meanwhile they shared the under-represented codons (UCG, GGG, GCG, CCG, CGG, ACG, CGA) and most ended with G except for CGA. Codon usage pattern of SARS-CoV-2 was generally found a high similarity to that of betacoronaviruses except for Bat coronavirus HKU4–1, Bat coronavirus HKU5–1(Fig. 4c, Supplementary Figures 5, 6, 7, 8). Moreover, the profiles of codon usage patterns among different genes of SARS-CoV-2 and non-human coronaviruses were further analyzed, as shown in Fig. 5 and Supplementary Figures 9, 10, 11, 12. We found similar codon usage pattern among SARS-CoV-2 and its phylogenetic relatives such as RaTG13, Bat-SL-CoVZC45, Bat-SL-CoVZXC21, PCoV_GX-P1E, PCoV_GX-P4L, which may reflect the evolutionary relationship between SARS-CoV-2 and these non-human coronaviruses. These results are in accordance with the full-genome phylogenetic analysis (Fig. 4a). The overall amino acid usage of the non-human coronaviruses was shown in Supplementary Figure 13. Similar to SARS-CoV-2, leucine and valine were the two most frequently used amino acids in all non-human coronaviruses analyzed in this study, CUU and GUU were preferred codons for leucine and valine, respectively.

Furthermore, similar to SARS-CoV-2, all the non-human coronaviruses analyzed in this study exhibited relatively low codon usage bias according to the intrinsic codon bias index (ICDI), codon bias index (CBI) and effective number of codons (ENC) values, as shown in Supplementary Figure 14. Nucleotide composition analysis (Supplementary Figure 4) and ENC-GC3S plot (Supplementary Figure 15) revealed that both mutational force and natural selection contribute to shape codon usage in non-human coronaviruses.

Overall, in the present study we attempted to take a snapshot of the characteristics of codon usage pattern in novel coronavirus SARS-CoV-2. As a result, we found all over-represented codons ended with A/U and this novel coronavirus had a relatively low codon usage bias. Both mutation pressure and natural selection were contributors to the bias. Additionally, the overall codon usage pattern of SARS-CoV-2 was generally similar to that of its phylogenetic relatives among non-human coronaviruses such as RaTG13. Our findings are consistent with the recent observations [11–15] and provide new insights into the characteristics of codon usage pattern in coronaviruses. These results also have important implications for future work.

Firstly, the information of genome-wide codon usage pattern of SARS-CoV-2 may be helpful to get new insights into the evolution of this newly emerging virus. With the increase of SARS-CoV-2 genome data available, we could reevaluate the codon usage pattern of SARS-CoV-2 more comprehensively to track the evolutionary changes between them. In this regard, genome-wide codon usage patterns in 100 complete genome sequences of SARS-CoV-2 isolates including SARS-CoV-2 Wuhan-Hu-1 from different geo-locations were analyzed herein. All information about the isolates can be found in Supplementary Table 3. The heatmap analysis (Supplementary Figure 16) revealed 12 preferred codons (GGU, GCU, UAA, GUU, UCU, CCU, ACU UAA, GGU, GCU, UCU, GUU, CCU, ACU) ending with A/U among all the 100 isolates, and the average RSCU value of these over-represented codons vary from 1.63 to 2.67 (Supplementary Figure 17). The highest RSCU value was for the codon AGA for R (2.67) amino acid and the lowest was UCG for S (0.11). We noted that the overall codon usage pattern appeared to be slightly variant among the tested 100 SARS-CoV-2 isolates from different geo-locations, reflecting minimal evolutionary changes among them.

Additionally, compared to other members of human coronaviruses as well as non-human coronaviruses in different hosts, we found that the overall codon usage pattern of SARS-CoV-2 is generally similar to that of its phylogenetic relatives among non-human betacoronaviruses such as RaTG13 (Fig. 5), which may reflect the evolutionary relationship between SARS-CoV-2 and these non-human coronaviruses.

Secondly, the information of genome-wide codon usage pattern of SARS-CoV-2 may have potential value for developing coronavirus vaccines to combat this pandemic disease. The information on codon usage by SARS-CoV-2 may pave the way to design strategies such as codon deoptimization [18–20], the use of the least preferred codons to modify the SARS-CoV-2 genome to reduce virulence for the development of a safe and effective vaccine. This strategy has several advantages. Deoptimized viruses could express an identical antigenic repertoire of T- and B-cell epitopes because they contain the intact wide type amino acid sequence. Moreover, deoptimized viruses can efficiently replicate in vitro while being highly attenuated in vivo, which is important for vaccine production and their safe implementation.

## Conclusions

Taking all these results together, our studies reveal that SARS-CoV-2 has a relatively low codon usage bias, which is shaped by both mutation pressure and natural selection. Additionally, there is slight variation in the codon usage pattern among the SARS-CoV-2 isolates from different geo-locations. Furthermore, the overall codon usage pattern of SARS-CoV-2 is generally similar to that of its phylogenetic relatives among non-human betacoronaviruses such as RaTG13. The information from this research may not only be helpful to get new insights into the evolution of human coronaviruses, but also have potential value for developing coronavirus vaccines.

## Supplementary information

**Additional file 1: Figure S1.** Nucleotide composition analysis of SARS-CoV-2 and other human coronaviruses.(A) Nucleotide frequency; (B) AU% and GC% content.

**Additional file 2: Figure S2.** Overall amino acid usage of SARS-CoV-2 and other human coronaviruses.

**Additional file 3: Figure S3.** ENC–GC3 plot. Effective number of codons (ENC) used in all human coronaviruses plotted against the GC3S, the GC content of synonymous codons at the third position. The orange curve plots the relationship between GC3S and ENC when codon usage bias is only affected by mutation pressure and in absence of selection. Red dots show the results obtained for SARS-CoV-2.

**Additional file 4: Figure S4.** Nucleotide composition analysis of SARS-CoV-2 and non-human coronaviruses.

**Additional file 5: Figure S5.** The profiles of the relative synonymous codon usage for complete gene of SARS-CoV-2 and non-human alphacoronavirus.

**Additional file 6: Figure S6.** The profiles of the relative synonymous codon usage for complete gene of SARS-CoV-2 and non-human betacoronavirus.

**Additional file 7: Figure S7.** The profiles of the relative synonymous codon usage for complete gene of SARS-CoV-2 and non-human gammacoronavirus.

**Additional file 8: Figure S8.** The profiles of the relative synonymous codon usage for complete gene of SARS-CoV-2 and non-human deltacoronavirus.

**Additional file 9: Figure S9.** The profiles of the relative synonymous codon usage for spike (S) gene of SARS-CoV-2 and non-human coronaviruses. Over-represented codons (RSCU value > 1.6) and under-represented codons (RSCU value < 0.6) were shown as line graph.

**Additional file 10: Figure S10.** The profiles of the relative synonymous codon usage for envelop (E) gene of SARS-CoV-2 and non-human coronaviruses. Over-represented codons (RSCU value > 1.6) and under-represented codons (RSCU value < 0.6) were shown as line graph.

**Additional file 11: Figure S11.** The profiles of the relative synonymous codon usage for membrane (M) gene of SARS-CoV-2 and non-human coronaviruses. Over-represented codons (RSCU value > 1.6) and under-represented codons (RSCU value < 0.6) were shown as line graph.

**Additional file 12: Figure S12.** The profiles of the relative synonymous codon usage for nucleocapsid (N) gene of SARS-CoV-2 and non-human coronaviruses. Over-represented codons (RSCU value > 1.6) and under-represented codons (RSCU value < 0.6) were shown as line graph.

**Additional file 13: Figure S13.** Overall amino acid usage of SARS-CoV-2 and non-human coronaviruses.

**Additional file 14: Figure S14.** The parameters of codon usage bias among SARS-CoV-2 and non-human coronaviruses analyzed in this study. ICDI: codon bias index; CBI: codon bias index; ENC: effective number of codons.

**Additional file 15: Figure S15.** ENC–GC3 plot. Effective number of codons (ENC) used in SARS-CoV-2 and all nonhuman coronaviruses plotted against the GC3S, the GC content of synonymous codons at the third position. The orange curve plots the relationship between GC3S and ENC when codon usage bias is only affected by mutation pressure and in absence of selection. Red dots show the results obtained for SARS-CoV-2.

**Additional file 16: Figure S16.** Heat map of RSCU values for 100 complete coding sequences of SARS-CoV-2. The heatmap analysis was performed using CIMminer. Each column represents a codon. Codons with higher RSCU values are highlighted with a red background.

**Additional file 17: Figure S17.** The average RSCU value of each codon for 100 complete genome of SARS-CoV-2 isolates.

**Additional file 18: Table S1.** Non-human coronaviruses analyzed in this study.

**Additional file 19: Table S2.** RSCU values in human coronaviruses analyzed in this study.

**Additional file 20: Table S3.** 100 complete genome of SARS-CoV-2 isolates analyzed in this study.

## Data Availability

This work was posted online in a preprint platform Research Square (https://www.researchsquare.com/article/rs-15071/v1; DOI: 10.21203/rs.2.24512/v1) on 26 Feb, 2020. The updated version is available from Research Square (https://www.researchsquare.com/article/rs-21553/v2; DOI: 10.21203/rs.3.rs-21553/v2).

## Abbreviations

COVID-19: Coronavirus disease 2019
CoVs: Coronaviruses
SARS-CoV: Severe acute respiratory syndrome coronavirus
MERS-CoV: Middle East respiratory syndrome coronavirus
SARS-CoV-2: Severe acute respiratory syndrome coronavirus 2
RSCU: Relative synonymous codon usage
ICDI: Intrinsic codon bias index
CBI: Codon bias index
ENC: Effective number of codons
