# Supplementary figures and images for: Characterization of codon usage pattern in SARS-CoV-2
Source: Virol J. 2020 Sep 14;17:138. doi: 10.1186/s12985-020-01395-x (PMC7487440; doi:10.1186/s12985-020-01395-x)

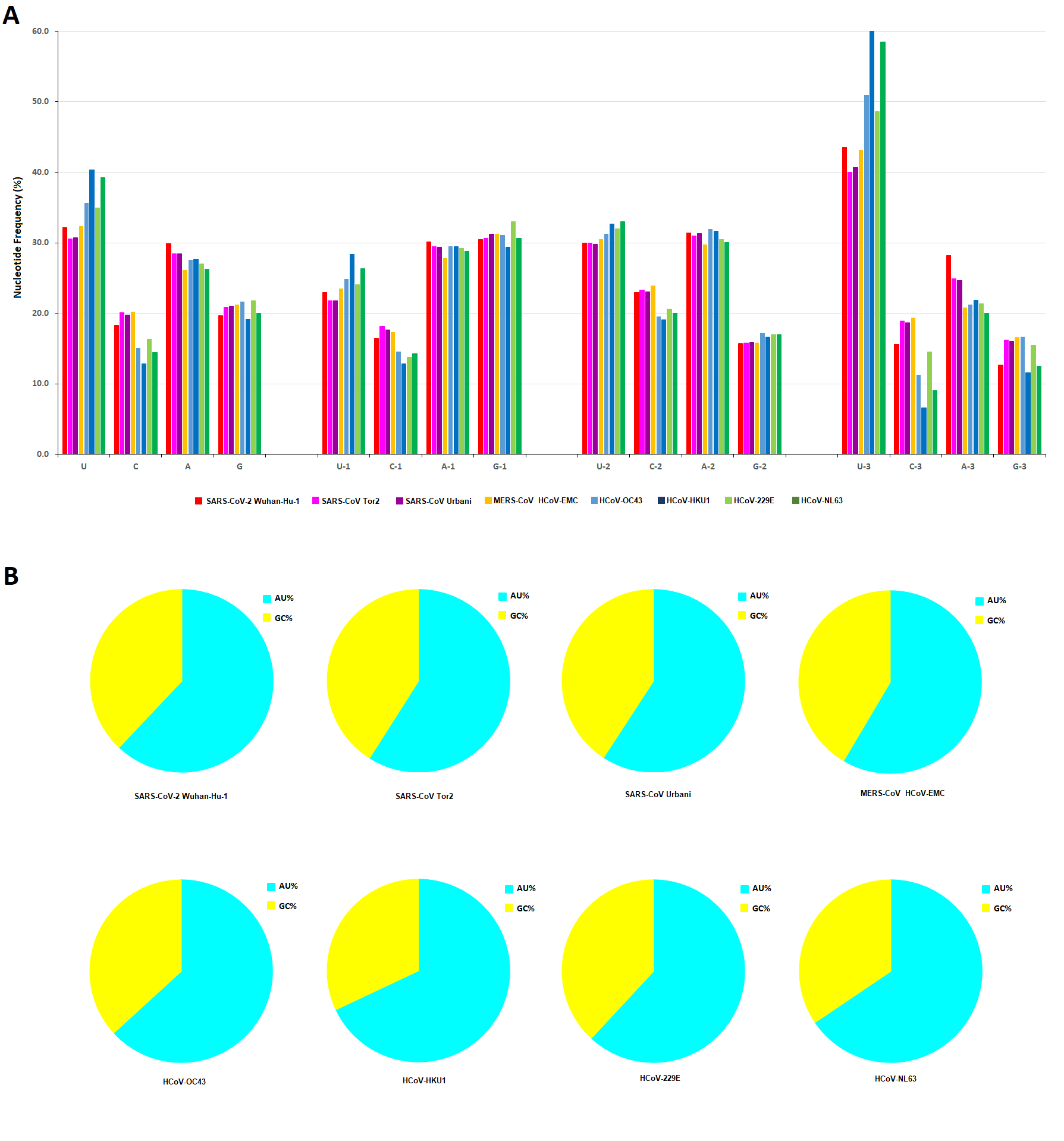

Supplement: Supplementary file 1 — Additional file 1: Figure S1. Nucleotide composition analysis of SARS-CoV-2 and other human coronaviruses.(A) Nucleotide frequency; (B) AU% and GC% content. [file 12985_2020_1395_MOESM1_ESM.tif]

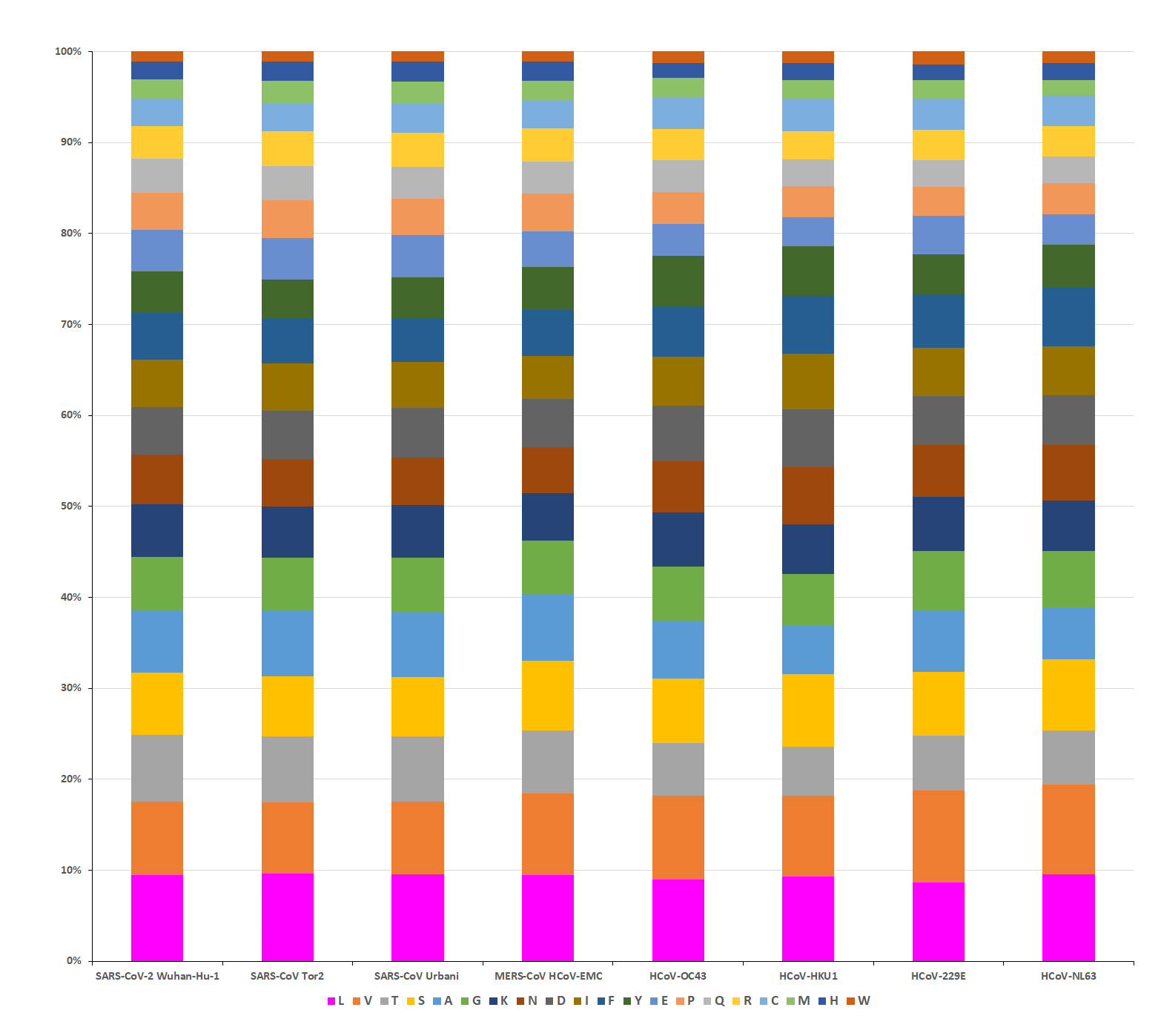

Supplement: Supplementary file 2 — Additional file 2: Figure S2. Overall amino acid usage of SARS-CoV-2 and other human coronaviruses. [file 12985_2020_1395_MOESM2_ESM.png]

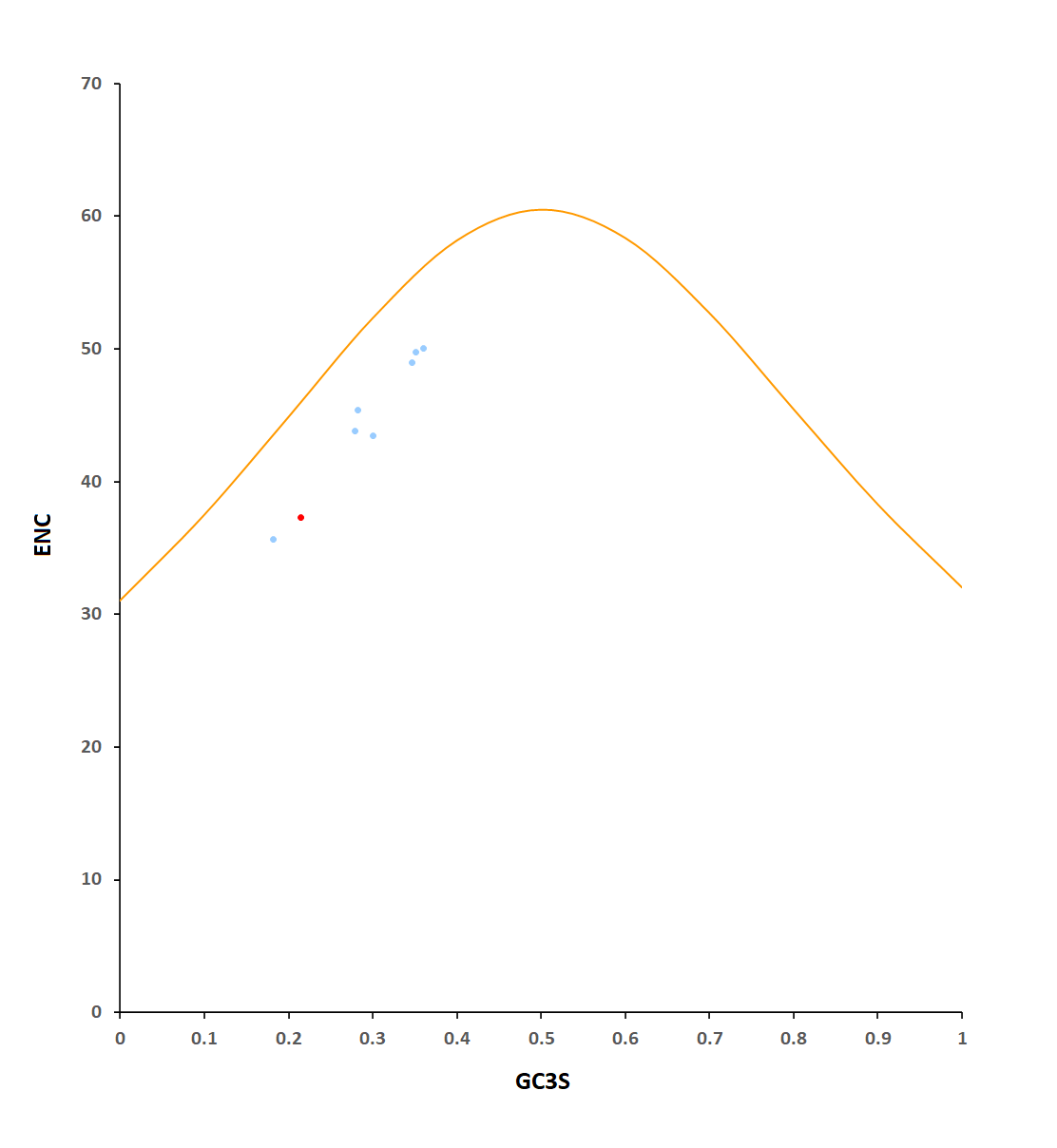

Supplement: Supplementary file 3 — Additional file 3: Figure S3. ENC–GC3 plot. Effective number of codons (ENC) used in all human coronaviruses plotted against the GC3S, the GC content of synonymous codons at the third position. The orange curve plots the relationship between GC3S and ENC when codon usage bias is only affected by mutation pressure and in absence of selection. Red dots show the results obtained for SARS-CoV-2. [file 12985_2020_1395_MOESM3_ESM.png]

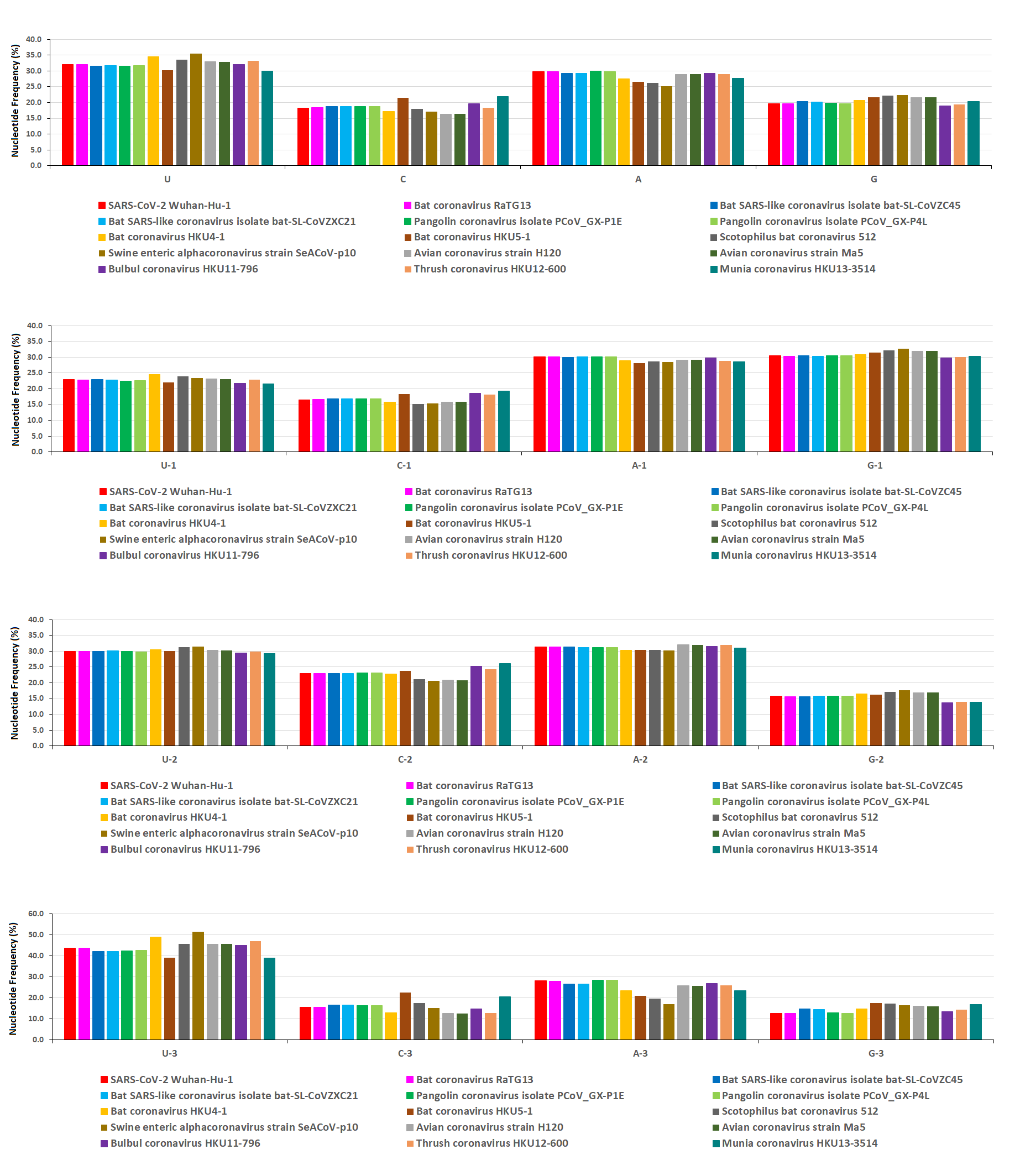

Supplement: Supplementary file 4 — Additional file 4: Figure S4. Nucleotide composition analysis of SARS-CoV-2 and non-human coronaviruses. [file 12985_2020_1395_MOESM4_ESM.tif]

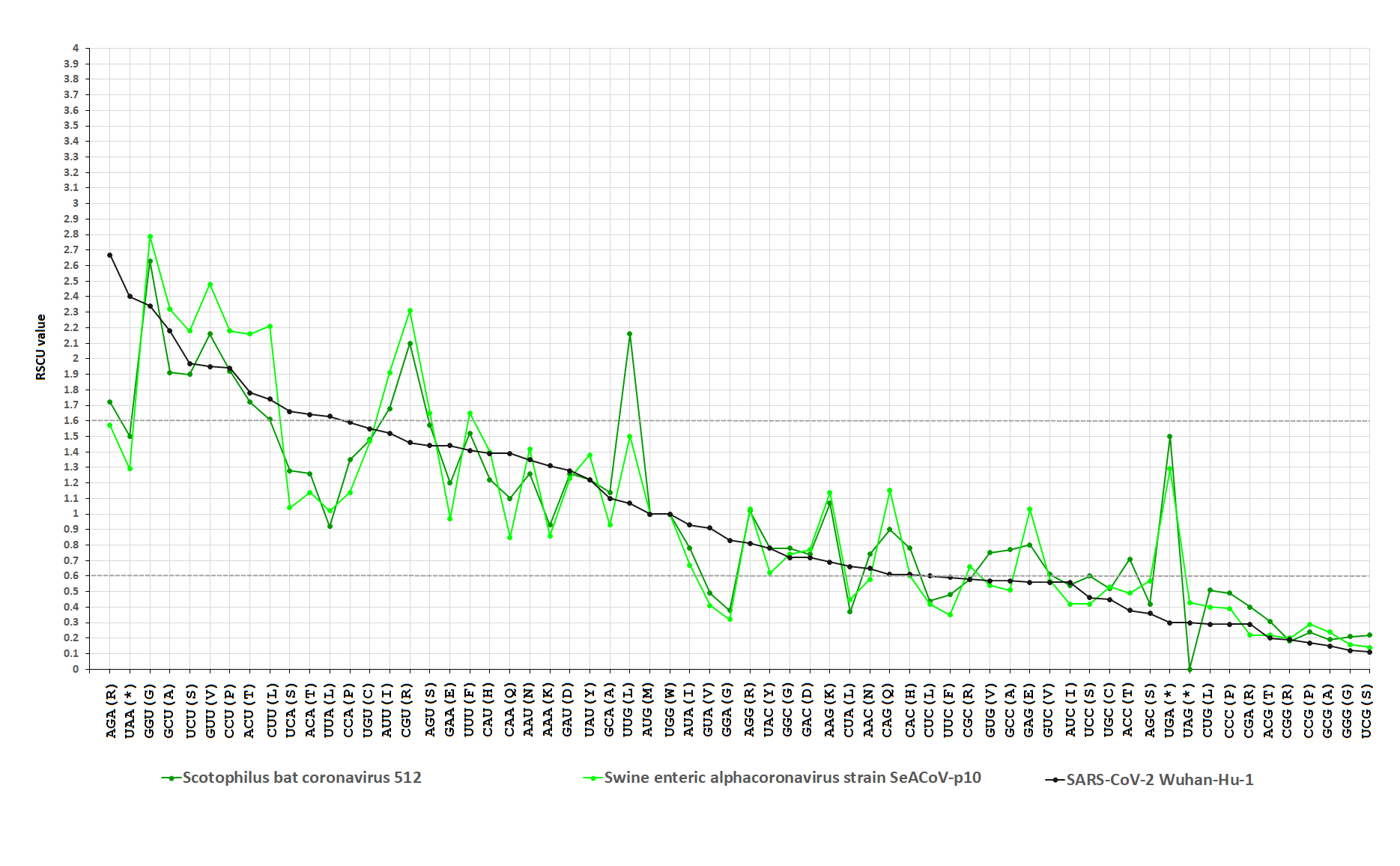

Supplement: Supplementary file 5 — Additional file 5: Figure S5. The profiles of the relative synonymous codon usage for complete gene of SARS-CoV-2 and non-human alphacoronavirus. [file 12985_2020_1395_MOESM5_ESM.tif]

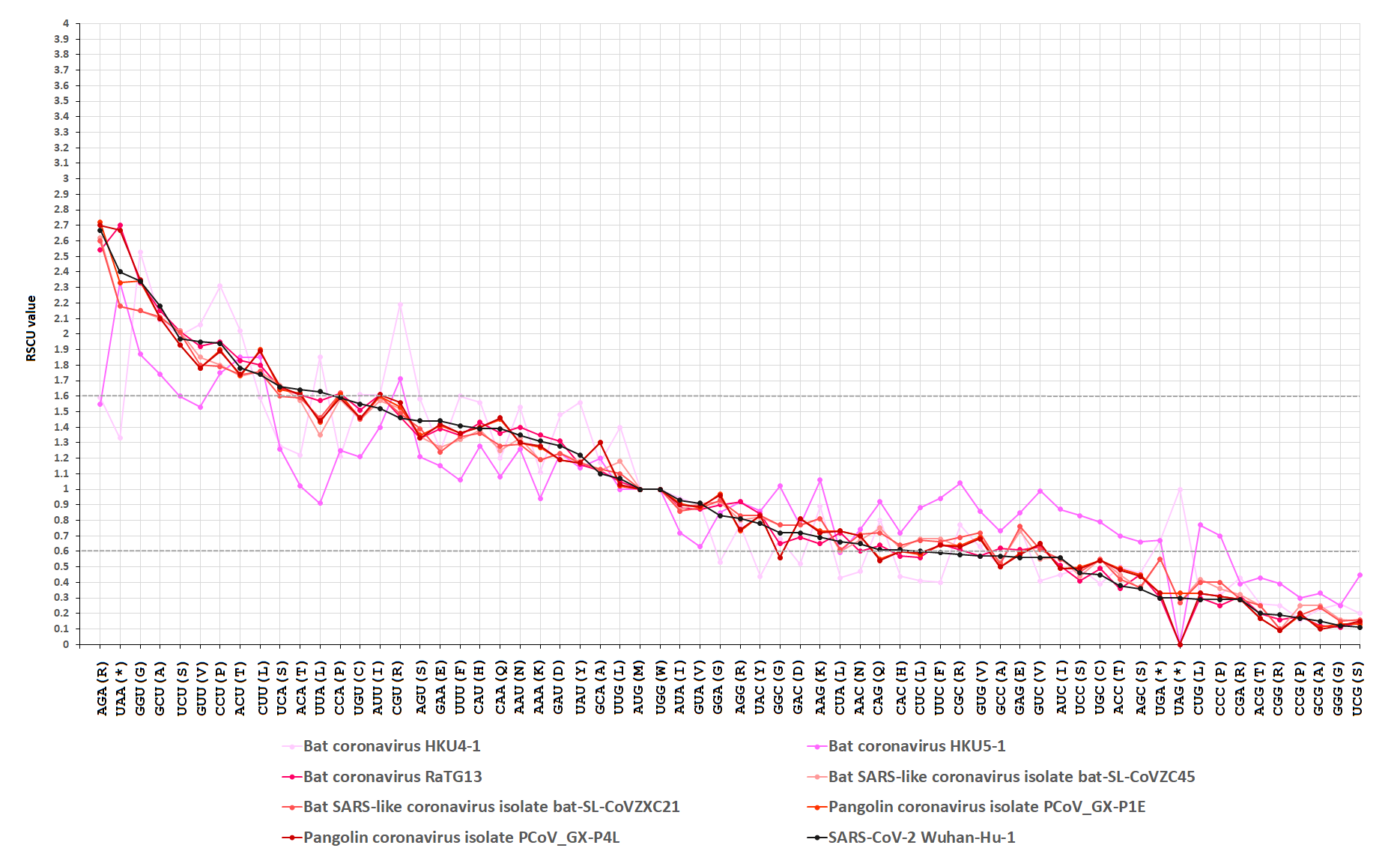

Supplement: Supplementary file 6 — Additional file 6: Figure S6. The profiles of the relative synonymous codon usage for complete gene of SARS-CoV-2 and non-human betacoronavirus. [file 12985_2020_1395_MOESM6_ESM.tif]

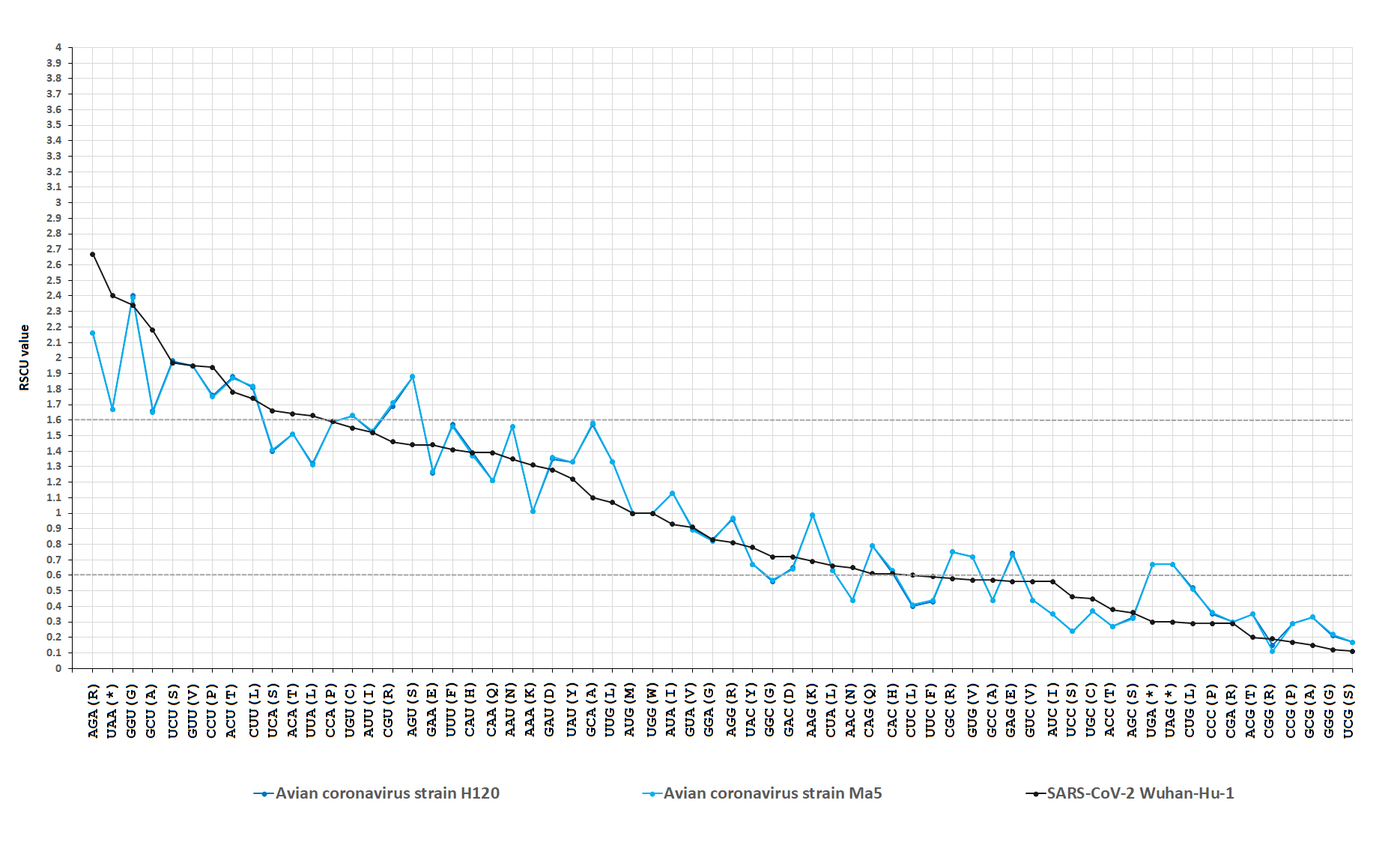

Supplement: Supplementary file 7 — Additional file 7: Figure S7. The profiles of the relative synonymous codon usage for complete gene of SARS-CoV-2 and non-human gammacoronavirus. [file 12985_2020_1395_MOESM7_ESM.tif]

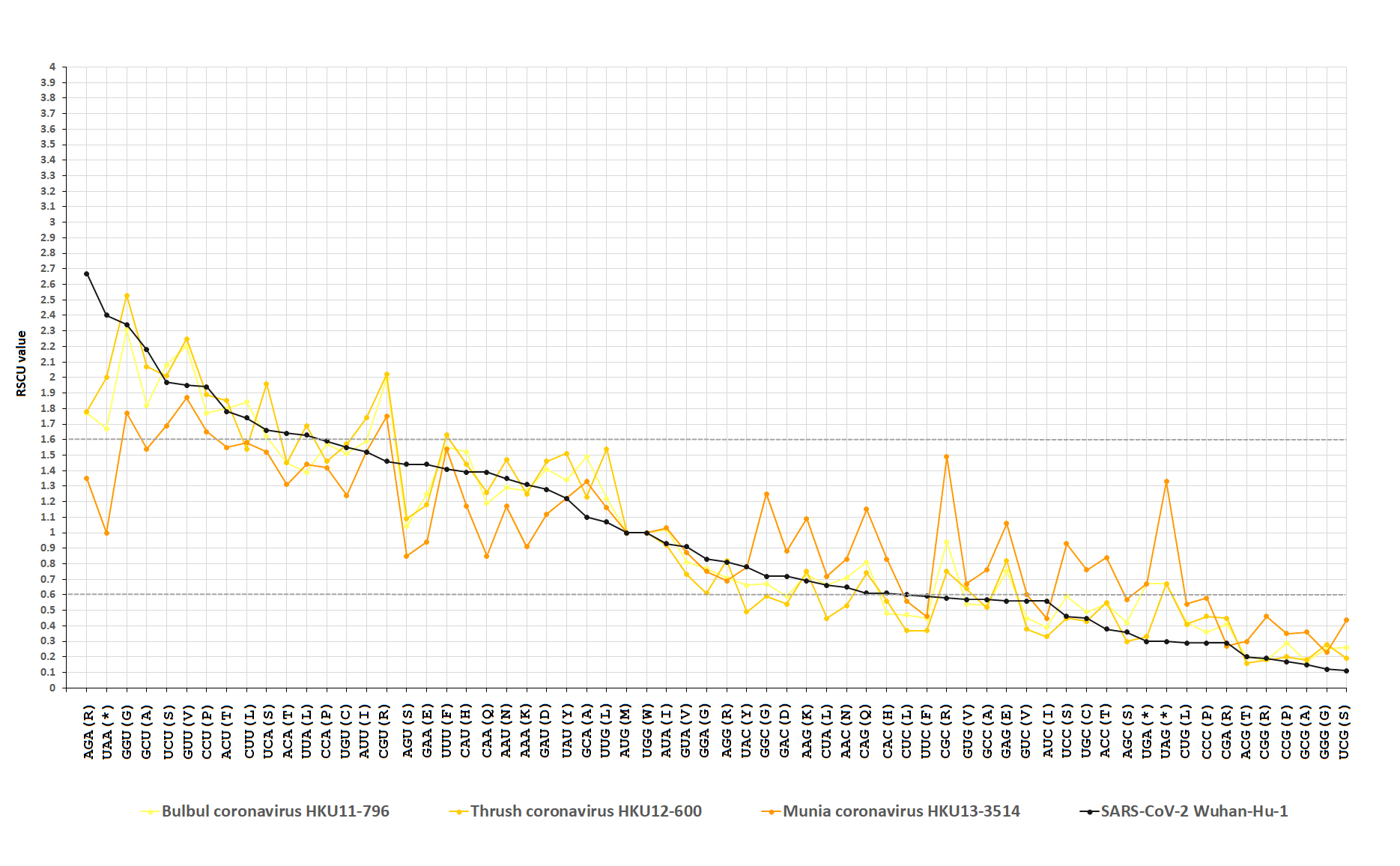

Supplement: Supplementary file 8 — Additional file 8: Figure S8. The profiles of the relative synonymous codon usage for complete gene of SARS-CoV-2 and non-human deltacoronavirus. [file 12985_2020_1395_MOESM8_ESM.tif]

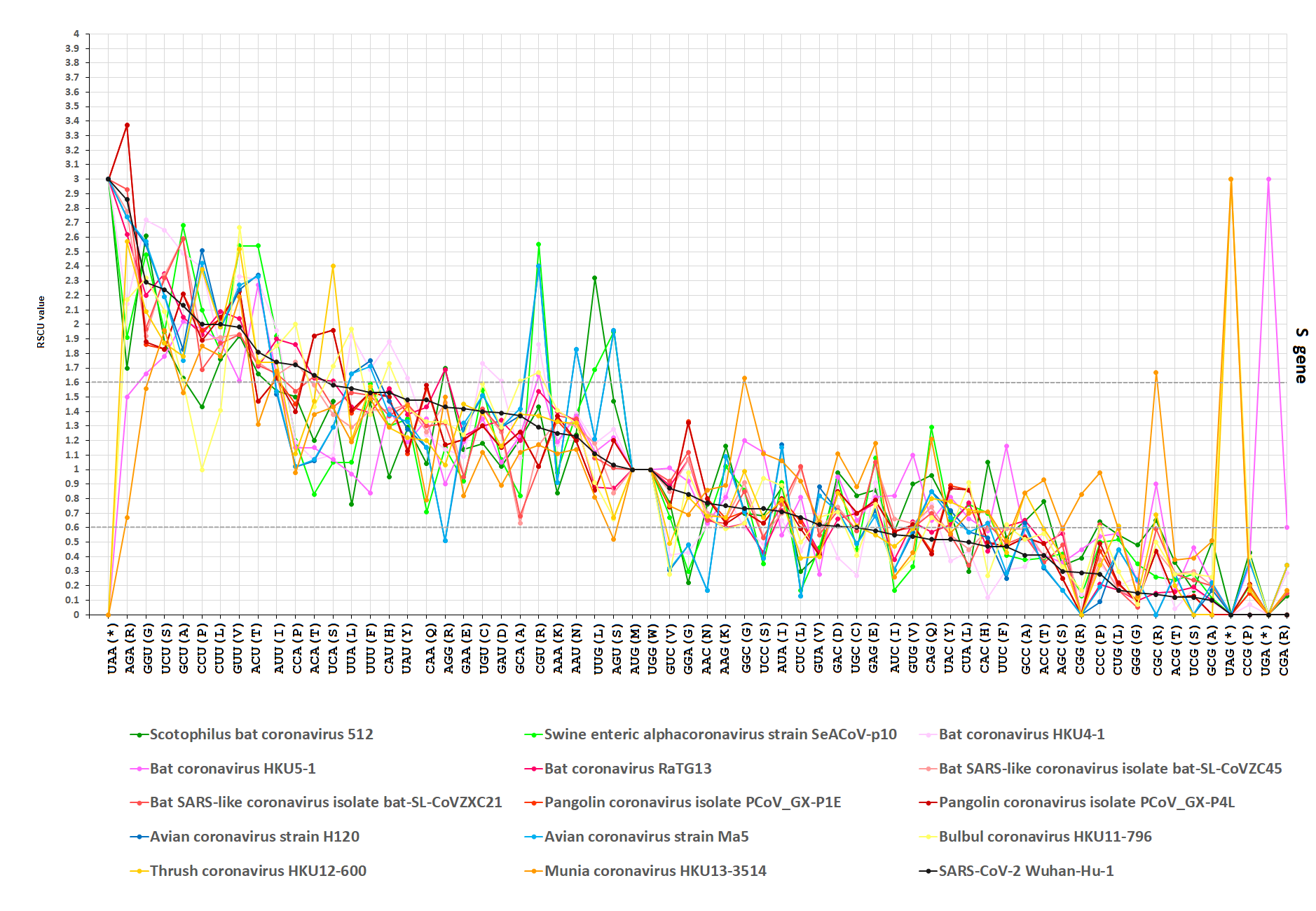

Supplement: Supplementary file 9 — Additional file 9: Figure S9. The profiles of the relative synonymous codon usage for spike (S) gene of SARS-CoV-2 and non-human coronaviruses. Over-represented codons (RSCU value > 1.6) and under-represented codons (RSCU value < 0.6) were shown as line graph. [file 12985_2020_1395_MOESM9_ESM.tif]

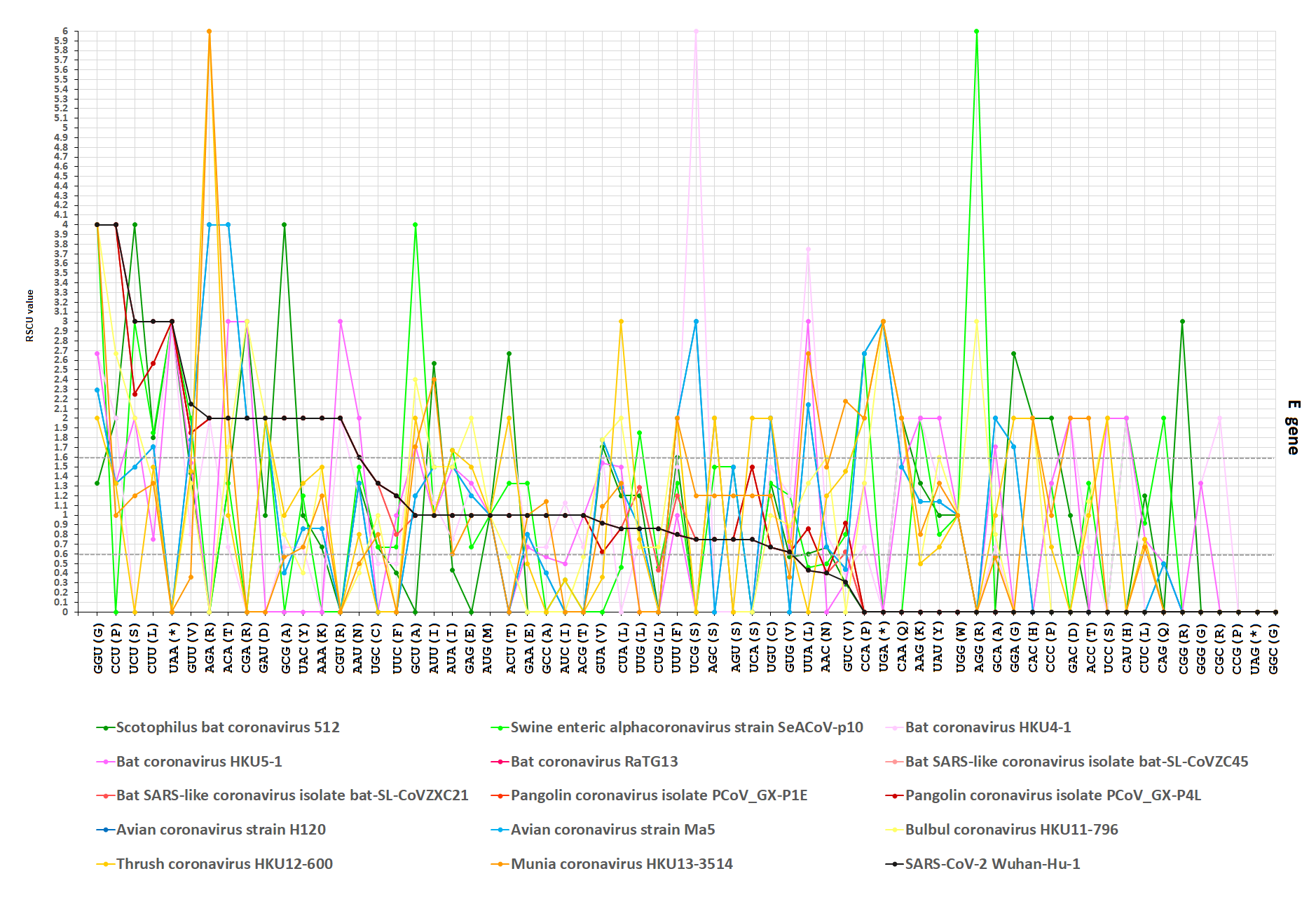

Supplement: Supplementary file 10 — Additional file 10: Figure S10. The profiles of the relative synonymous codon usage for envelop (E) gene of SARS-CoV-2 and non-human coronaviruses. Over-represented codons (RSCU value > 1.6) and under-represented codons (RSCU value < 0.6) were shown as line graph. [file 12985_2020_1395_MOESM10_ESM.tif]

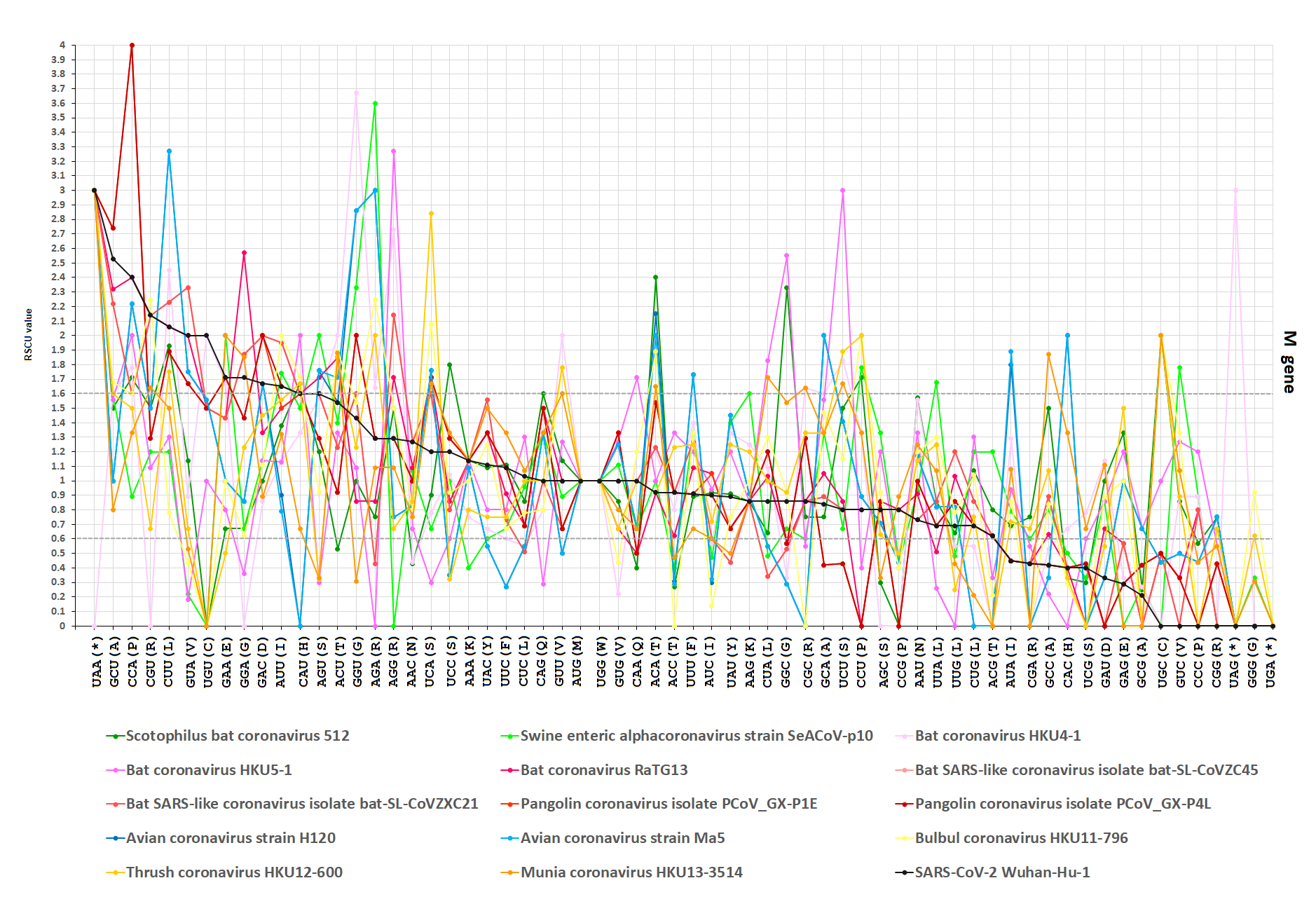

Supplement: Supplementary file 11 — Additional file 11: Figure S11. The profiles of the relative synonymous codon usage for membrane (M) gene of SARS-CoV-2 and non-human coronaviruses. Over-represented codons (RSCU value > 1.6) and under-represented codons (RSCU value < 0.6) were shown as line graph. [file 12985_2020_1395_MOESM11_ESM.tif]

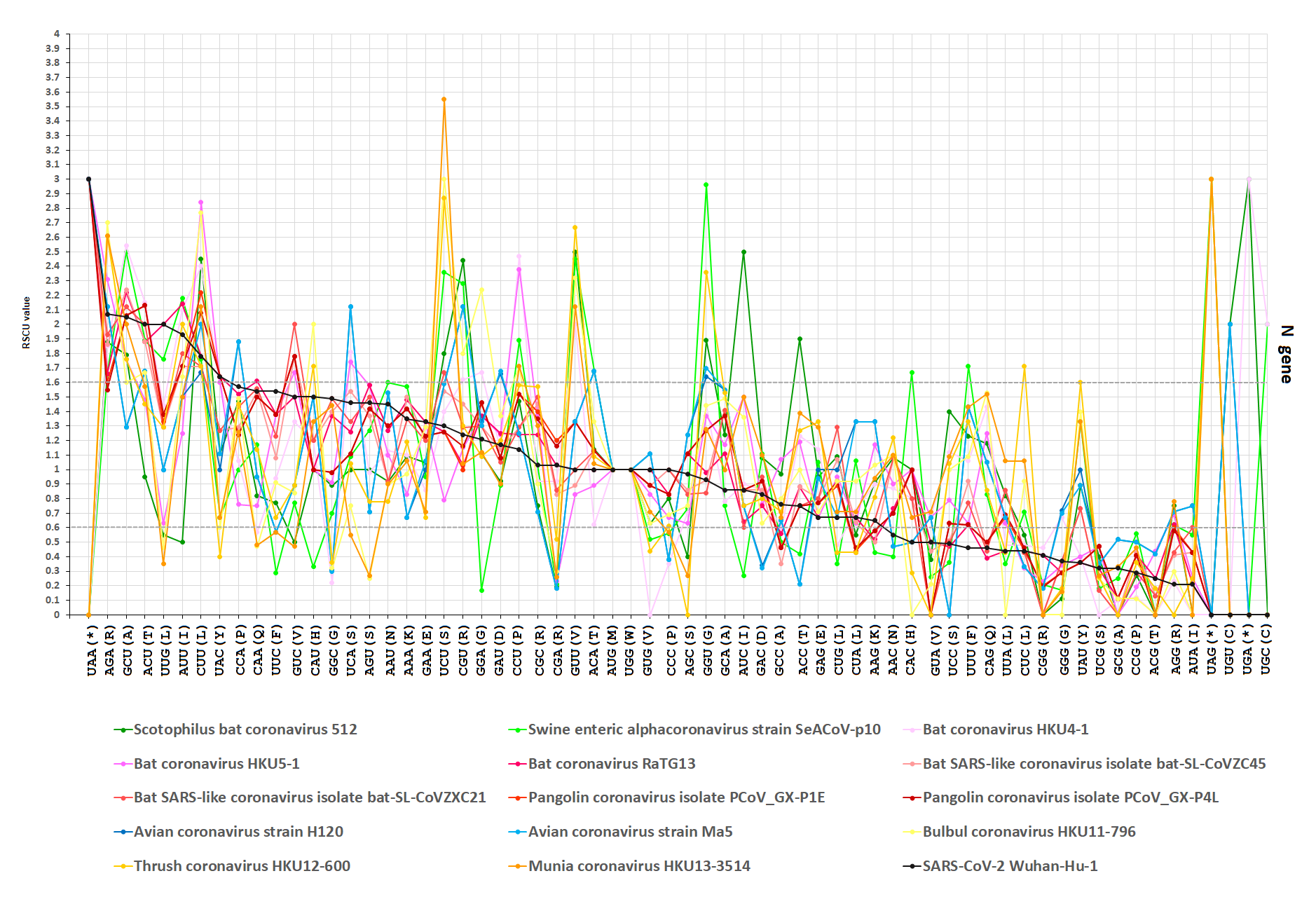

Supplement: Supplementary file 12 — Additional file 12: Figure S12. The profiles of the relative synonymous codon usage for nucleocapsid (N) gene of SARS-CoV-2 and non-human coronaviruses. Over-represented codons (RSCU value > 1.6) and under-represented codons (RSCU value < 0.6) were shown as line graph. [file 12985_2020_1395_MOESM12_ESM.tif]

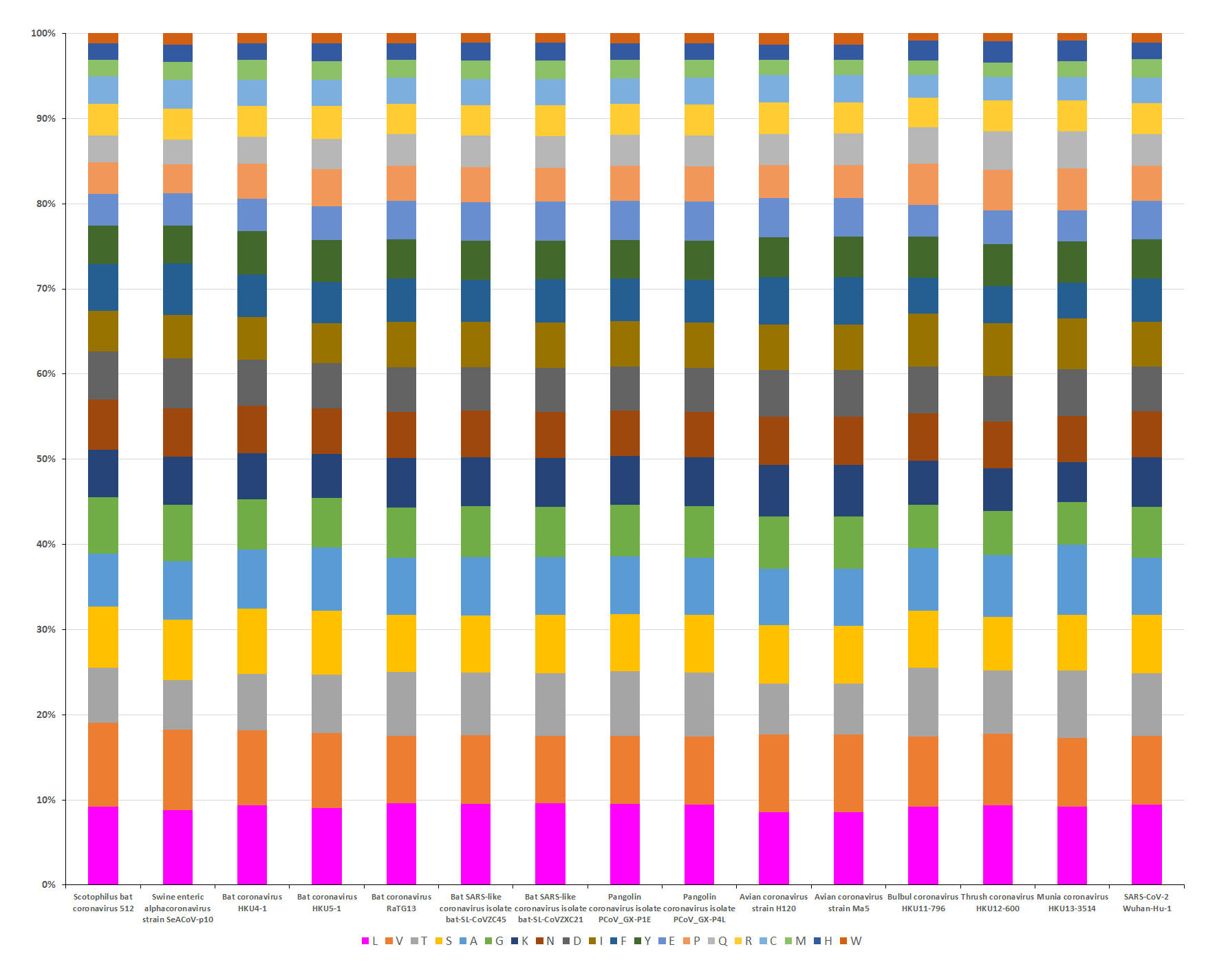

Supplement: Supplementary file 13 — Additional file 13: Figure S13. Overall amino acid usage of SARS-CoV-2 and non-human coronaviruses. [file 12985_2020_1395_MOESM13_ESM.png]

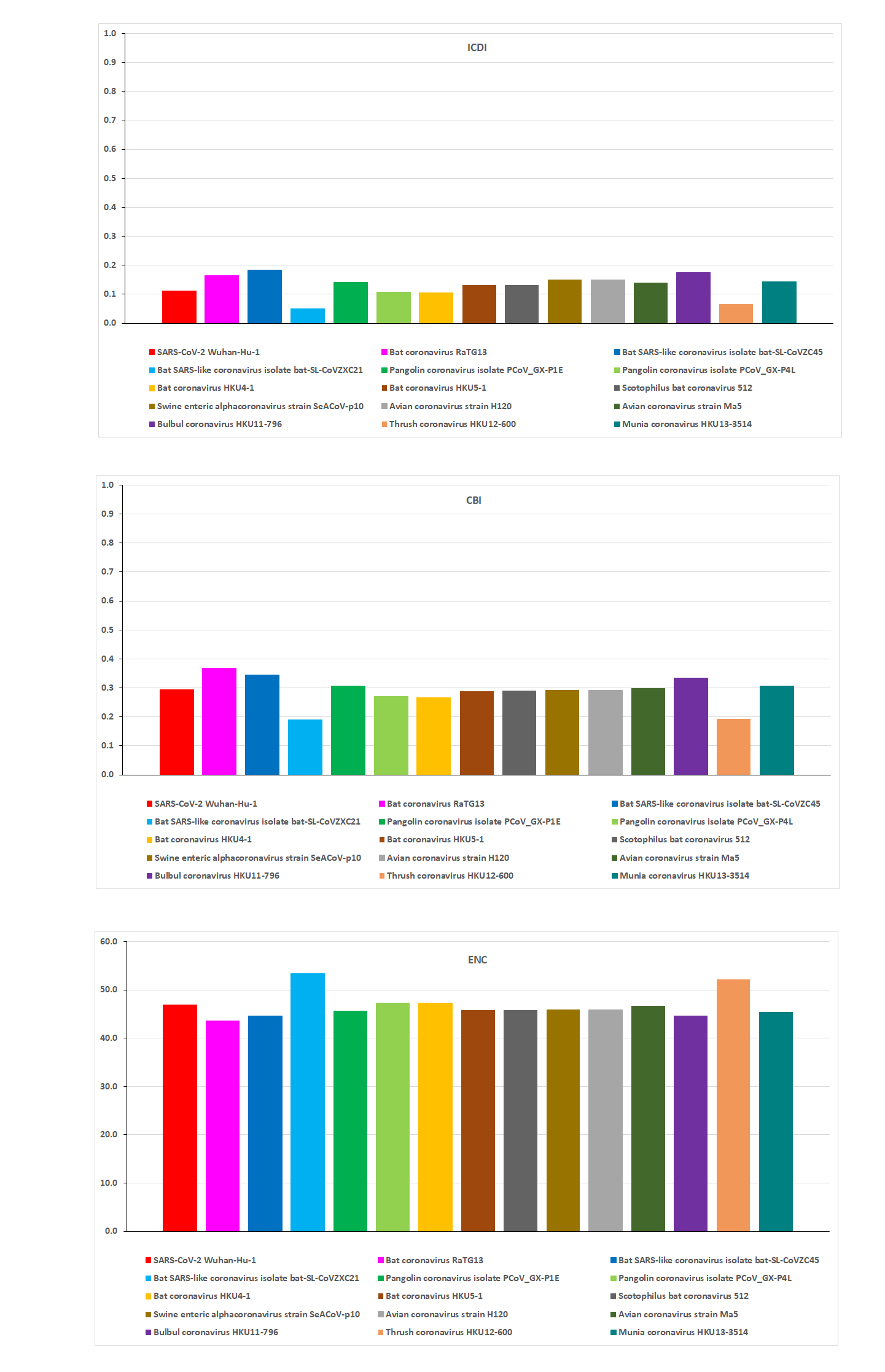

Supplement: Supplementary file 14 — Additional file 14: Figure S14. The parameters of codon usage bias among SARS-CoV-2 and non-human coronaviruses analyzed in this study. ICDI: codon bias index; CBI: codon bias index; ENC: effective number of codons. [file 12985_2020_1395_MOESM14_ESM.tif]

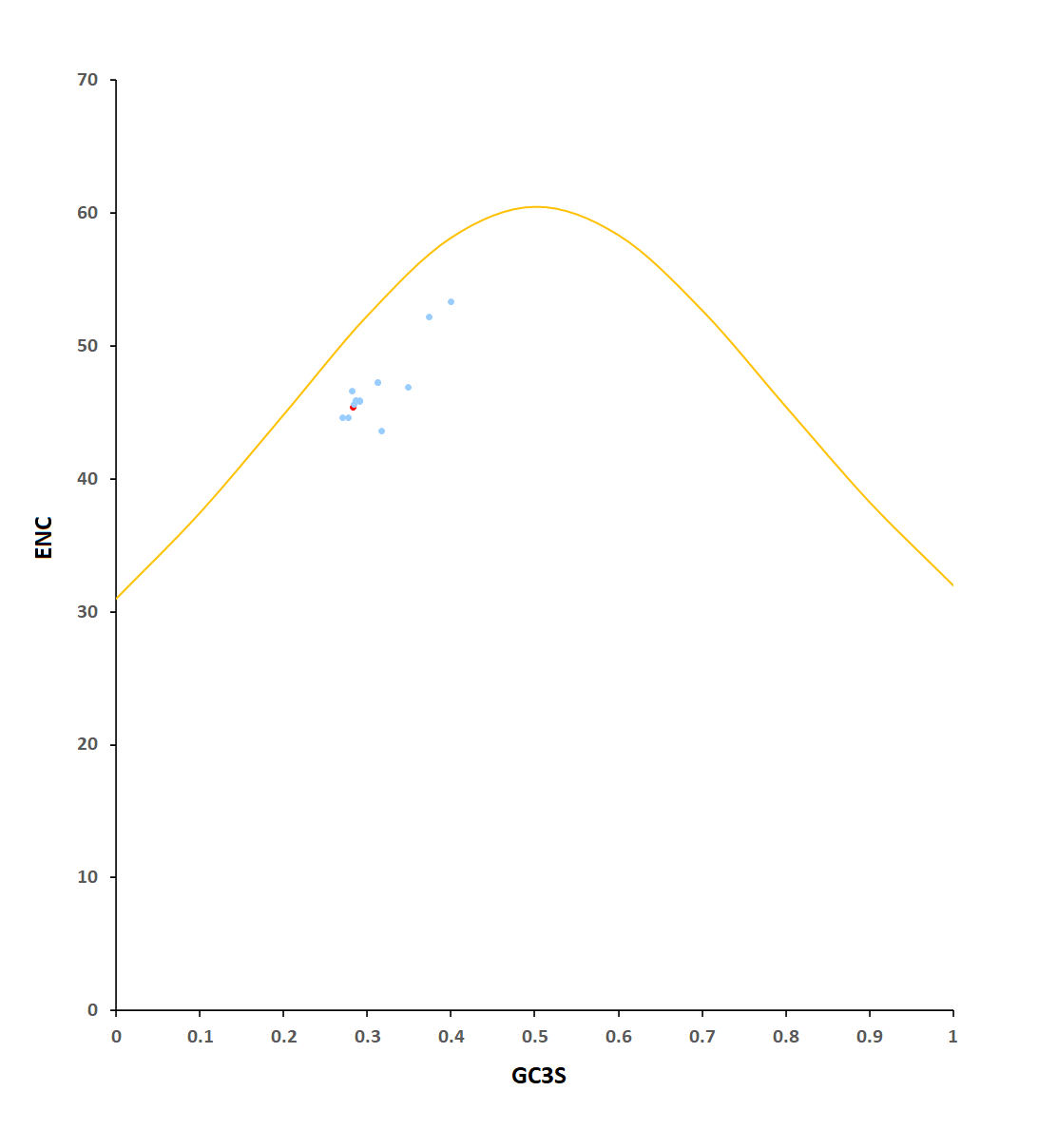

Supplement: Supplementary file 15 — Additional file 15: Figure S15. ENC–GC3 plot. Effective number of codons (ENC) used in SARS-CoV-2 and all nonhuman coronaviruses plotted against the GC3S, the GC content of synonymous codons at the third position. The orange curve plots the relationship between GC3S and ENC when codon usage bias is only affected by mutation pressure and in absence of selection. Red dots show the results obtained for SARS-CoV-2. [file 12985_2020_1395_MOESM15_ESM.png]

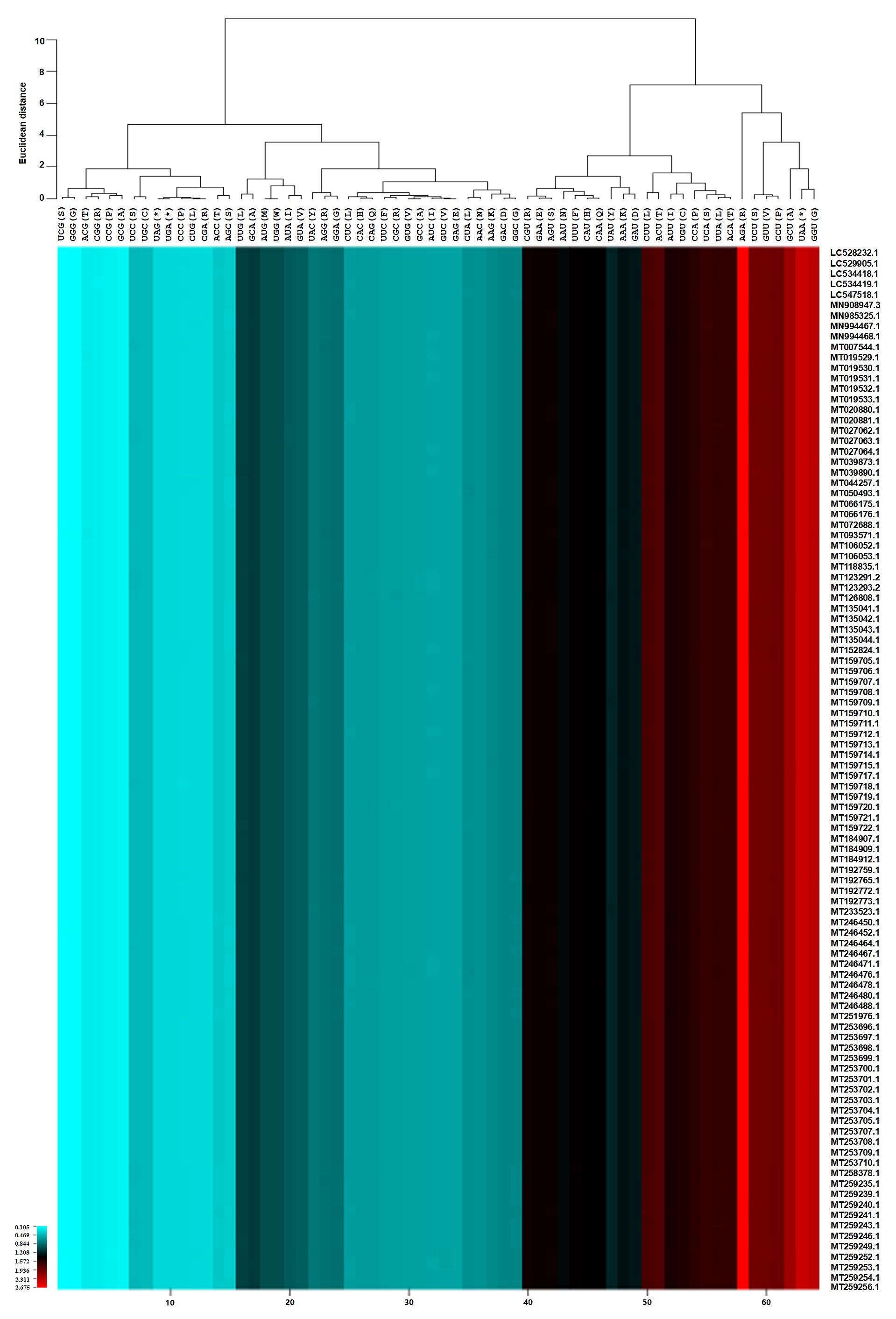

Supplement: Supplementary file 16 — Additional file 16: Figure S16. Heat map of RSCU values for 100 complete coding sequences of SARS-CoV-2. The heatmap analysis was performed using CIMminer. Each column represents a codon. Codons with higher RSCU values are highlighted with a red background. [file 12985_2020_1395_MOESM16_ESM.tif]

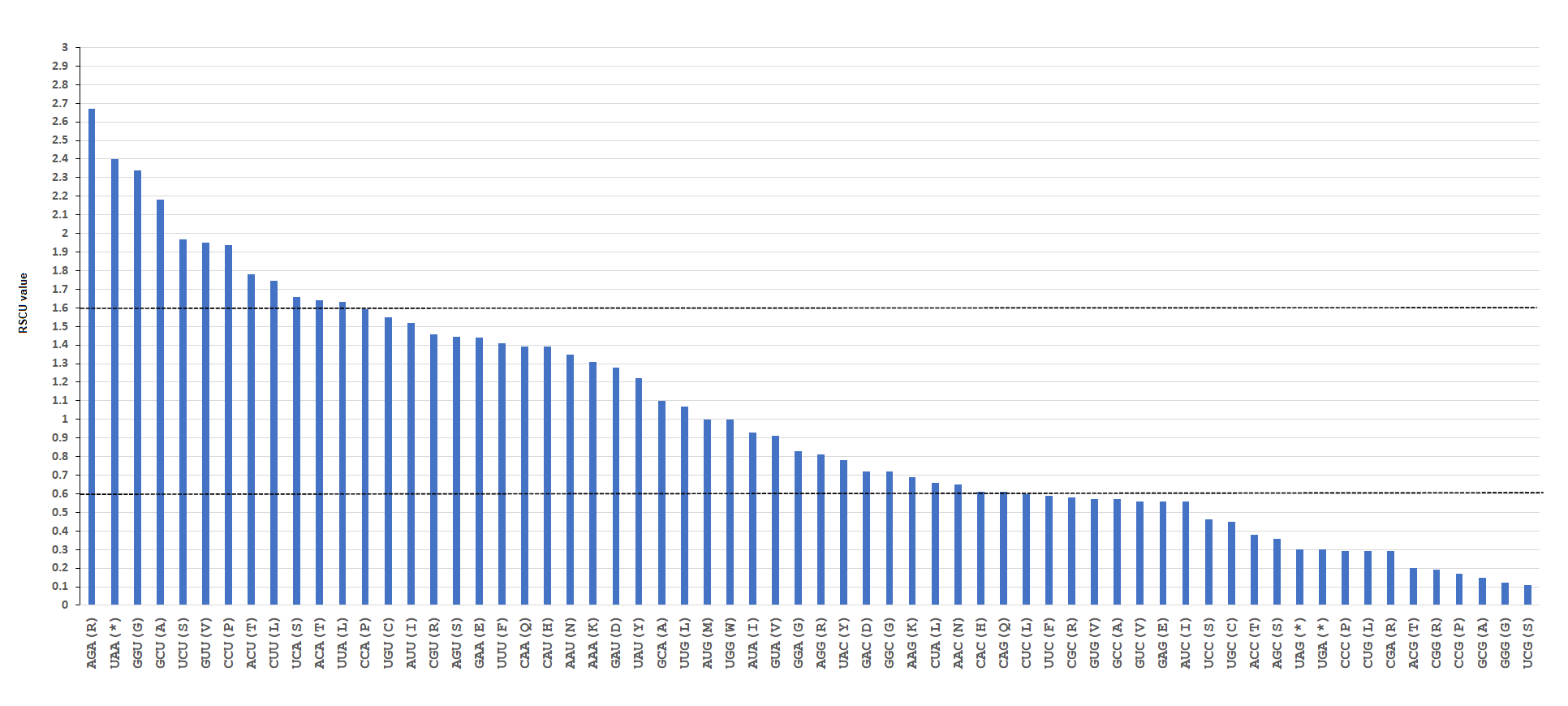

Supplement: Supplementary file 17 — Additional file 17: Figure S17. The average RSCU value of each codon for 100 complete genome of SARS-CoV-2 isolates. [file 12985_2020_1395_MOESM17_ESM.tif]
