# Supplementary material for: Characterization of codon usage pattern in SARS-CoV-2
Source: Virol J. 2020 Sep 14;17:138. doi: 10.1186/s12985-020-01395-x (PMC7487440; doi:10.1186/s12985-020-01395-x)
Supplement: Supplementary file 18 — Additional file 18: Table S1. Non-human coronaviruses analyzed in this study. [file 12985_2020_1395_MOESM18_ESM.docx]

**Supplementary Table 1 Non-human coronaviruses analyzed in this study**.

|  | Coronavirus | Strain | Host | ACCESSION | Genome Length(nt) |
| --- | --- | --- | --- | --- | --- |
| Alphacoronavirus | Scotophilus bat coronavirus 512 | BtCoV/512/2005 | Lesser Asiatic yellow house bat | NC_009657.1 | 28203 |
|  | Swine enteric alphacoronavirus strain SeACoV-p10 | SeACoV-p10 | Swine | MK977618.1 | 27155 |
| Betacoronavirus | Bat coronavirus HKU4-1 | HKU4-1 B04f | Lesser bamboo bat | NC_009019.1 | 30286 |
|  | Bat coronavirus HKU5-1 | HKU5-1 LMH03f | Japanese pipistrelle bat | NC_009020.1 | 30482 |
|  | Bat coronavirus RaTG13 | RaTG13 | Rhinolophus affinis bat | MN996532.1 | 29855 |
|  | Bat SARS-like coronavirus isolate bat-SL-CoVZC45 | Bat-SL-CoVZC45 | Rhinolophus sinicus | MG772933.1 | 29802 |
|  | Bat SARS-like coronavirus isolate bat-SL-CoVZXC21 | Bat-SL-CoVZXC21 | Rhinolophus sinicus | MG772934.1 | 29732 |
|  | Pangolin coronavirus isolate PCoV_GX-P1E | PCoV_GX-P1E | Manis javanica (Malayan pangolin) | MT040334.1 | 29801 |
|  | Pangolin coronavirus isolate PCoV_GX-P4L | PCoV_GX-P4L | Manis javanica (Malayan pangolin) | MT040333.1 | 29805 |
| Gammacoronavirus | Avian coronavirus strain H120 | H120 | Gallus gallus | MK071267.1 | 27632 |
|  | Avian coronavirus strain Ma5 | Ma5 | Chicken | KY626045.1 | 27652 |
| Deltacoronavirus | Bulbul coronavirus HKU11-796 | HKU11-796 | Chinese bulbul | FJ376620.1 | 26476 |
|  | Thrush coronavirus HKU12-600 | ThCoV HKU12-600 | Gray-backed thrush | NC_011549.1 | 26396 |
|  | Munia coronavirus HKU13-3514 | MuCoV HKU13-3514 | White-rumped munia | NC_011550.1 | 26552 |
