# Supplementary material for: Characterization of codon usage pattern in SARS-CoV-2
Source: Virol J. 2020 Sep 14;17:138. doi: 10.1186/s12985-020-01395-x (PMC7487440; doi:10.1186/s12985-020-01395-x)
Supplement: Supplementary file 19 — Additional file 19: Table S2. RSCU values in human coronaviruses analyzed in this study. [file 12985_2020_1395_MOESM19_ESM.docx]

**Supplementary Table 2 RSCU values in human coronaviruses analyzed in this study.**

|  | **SARS-CoV-2 Wuhan-Hu-1** | **SARS-CoV Tor2** | **SARS-CoV Urbani** | **MERS-CoV HCoV-EMC** | **HCoV-OC43** | **HCoV-HKU1** | **HCoV-229E** | **HCoV-NL63** |
| --- | --- | --- | --- | --- | --- | --- | --- | --- |
| AGA(R) | 2.67 | 2.09 | 2.16 | 1.32 | 1.83 | 2.06 | 2.23 | 1.41 |
| UAA(*) | 2.4 | 2.14 | 2.75 | 2.18 | 1.88 | 1.71 | 2.63 | 2 |
| GGU(G) | 2.34 | 1.91 | 1.99 | 2.05 | 2.44 | 3.08 | 2.72 | 3.31 |
| GCU(A) | 2.18 | 2.06 | 2.09 | 2.06 | 2.15 | 2.66 | 2.15 | 2.38 |
| UCU(S) | 1.97 | 1.92 | 1.93 | 2.09 | 1.81 | 2.73 | 2.16 | 2.36 |
| GUU(V) | 1.95 | 1.67 | 1.68 | 1.78 | 2.21 | 2.73 | 2.33 | 2.91 |
| CCU(P) | 1.94 | 1.72 | 1.73 | 1.95 | 2.06 | 2.65 | 2.04 | 2.46 |
| ACU(T) | 1.78 | 1.7 | 1.7 | 1.94 | 1.97 | 2.65 | 1.94 | 2.41 |
| CUU(L) | 1.74 | 1.77 | 1.79 | 1.69 | 1.49 | 1.34 | 1.74 | 1.86 |
| UCA(S) | 1.66 | 1.77 | 1.76 | 1.21 | 0.88 | 0.85 | 1.07 | 1.15 |
| ACA(T) | 1.64 | 1.55 | 1.6 | 1.18 | 1.28 | 1.01 | 1.46 | 1.16 |
| UUA(L) | 1.63 | 1.04 | 1.08 | 1.21 | 1.49 | 2.45 | 1.11 | 1.68 |
| CCA(P) | 1.59 | 1.69 | 1.75 | 1.22 | 1.22 | 0.93 | 1.32 | 1.21 |
| UGU(C) | 1.55 | 1.23 | 1.28 | 1.18 | 1.51 | 1.8 | 1.46 | 1.82 |
| AUU(I) | 1.52 | 1.7 | 1.67 | 1.71 | 1.74 | 2.01 | 1.88 | 2.09 |
| CGU(R) | 1.46 | 1.57 | 1.7 | 1.79 | 1.99 | 2.35 | 2.05 | 2.85 |
| AGU(S) | 1.44 | 1.13 | 1.2 | 1.29 | 2.1 | 1.94 | 1.59 | 1.91 |
| GAA(E) | 1.44 | 1.06 | 1.06 | 1.05 | 1.19 | 1.4 | 1.39 | 1.32 |
| UUU(F) | 1.41 | 1.24 | 1.24 | 1.28 | 1.75 | 1.85 | 1.63 | 1.79 |
| CAU(H) | 1.39 | 1.29 | 1.3 | 1.32 | 1.57 | 1.73 | 1.52 | 1.59 |
| CAA(Q) | 1.39 | 1.21 | 1.17 | 1.14 | 1.08 | 1.37 | 1.33 | 1.31 |
| AAU(N) | 1.35 | 1.25 | 1.26 | 1.4 | 1.66 | 1.76 | 1.39 | 1.58 |
| AAA(K) | 1.31 | 1.06 | 1.05 | 1.01 | 1.02 | 1.34 | 1.13 | 1.18 |
| GAU(D) | 1.28 | 1.25 | 1.27 | 1.27 | 1.64 | 1.71 | 1.26 | 1.56 |
| UAU(Y) | 1.22 | 1.12 | 1.13 | 1.27 | 1.66 | 1.81 | 1.34 | 1.62 |
| GCA(A) | 1.1 | 1.11 | 1.12 | 1 | 1.1 | 0.93 | 1.12 | 1.06 |
| UUG(L) | 1.07 | 1.06 | 1.09 | 1.43 | 1.9 | 1.68 | 2.04 | 1.85 |
| AUG(M) | 1 | 1 | 1 | 1 | 1 | 1 | 1 | 1 |
| UGG(W) | 1 | 1 | 1 | 1 | 1 | 1 | 1 | 1 |
| AUA(I) | 0.93 | 0.67 | 0.65 | 0.71 | 0.96 | 0.86 | 0.74 | 0.72 |
| GUA(V) | 0.91 | 0.86 | 0.87 | 0.72 | 0.7 | 0.77 | 0.44 | 0.42 |
| GGA(G) | 0.83 | 0.9 | 0.89 | 0.65 | 0.68 | 0.4 | 0.4 | 0.29 |
| AGG(R) | 0.81 | 0.99 | 0.9 | 0.88 | 0.7 | 0.55 | 0.63 | 0.85 |
| UAC(Y) | 0.78 | 0.88 | 0.87 | 0.73 | 0.34 | 0.19 | 0.66 | 0.38 |
| GGC(G) | 0.72 | 1.01 | 0.96 | 1 | 0.64 | 0.37 | 0.77 | 0.3 |
| GAC(D) | 0.72 | 0.75 | 0.73 | 0.73 | 0.36 | 0.29 | 0.74 | 0.44 |
| AAG(K) | 0.69 | 0.94 | 0.95 | 0.99 | 0.98 | 0.66 | 0.87 | 0.82 |
| CUA(L) | 0.66 | 0.7 | 0.65 | 0.48 | 0.39 | 0.23 | 0.39 | 0.24 |
| AAC(N) | 0.65 | 0.75 | 0.74 | 0.6 | 0.34 | 0.24 | 0.61 | 0.42 |
| CAG(Q) | 0.61 | 0.79 | 0.83 | 0.86 | 0.92 | 0.63 | 0.67 | 0.69 |
| CAC(H) | 0.61 | 0.71 | 0.7 | 0.68 | 0.43 | 0.27 | 0.48 | 0.41 |
| CUC(L) | 0.6 | 0.85 | 0.81 | 0.69 | 0.29 | 0.16 | 0.31 | 0.22 |
| UUC(F) | 0.59 | 0.76 | 0.76 | 0.72 | 0.25 | 0.15 | 0.37 | 0.21 |
| CGC(R) | 0.58 | 0.77 | 0.76 | 1.14 | 0.72 | 0.47 | 0.67 | 0.51 |
| GUG(V) | 0.57 | 0.78 | 0.77 | 0.74 | 0.77 | 0.26 | 0.74 | 0.31 |
| GCC(A) | 0.57 | 0.58 | 0.56 | 0.62 | 0.55 | 0.3 | 0.49 | 0.45 |
| GAG(E) | 0.56 | 0.94 | 0.94 | 0.95 | 0.81 | 0.6 | 0.61 | 0.68 |
| GUC(V) | 0.56 | 0.69 | 0.68 | 0.77 | 0.33 | 0.24 | 0.48 | 0.36 |
| AUC(I) | 0.56 | 0.63 | 0.68 | 0.59 | 0.29 | 0.13 | 0.38 | 0.19 |
| UCC(S) | 0.46 | 0.4 | 0.39 | 0.75 | 0.45 | 0.19 | 0.49 | 0.26 |
| UGC(C) | 0.45 | 0.77 | 0.72 | 0.82 | 0.49 | 0.2 | 0.54 | 0.18 |
| ACC(T) | 0.38 | 0.55 | 0.53 | 0.71 | 0.54 | 0.23 | 0.43 | 0.3 |
| AGC(S) | 0.36 | 0.54 | 0.5 | 0.46 | 0.57 | 0.19 | 0.54 | 0.27 |
| UAG(*) | 0.3 | 0.43 | 0 | 0.55 | 0.75 | 0.86 | 0.38 | 0 |
| UGA(*) | 0.3 | 0.43 | 0.25 | 0.27 | 0.38 | 0.43 | 0 | 1 |
| CCC(P) | 0.29 | 0.42 | 0.38 | 0.64 | 0.49 | 0.27 | 0.42 | 0.21 |
| CUG(L) | 0.29 | 0.59 | 0.58 | 0.5 | 0.44 | 0.15 | 0.4 | 0.13 |
| CGA(R) | 0.29 | 0.47 | 0.39 | 0.45 | 0.46 | 0.27 | 0.27 | 0.28 |
| ACG(T) | 0.2 | 0.2 | 0.18 | 0.17 | 0.22 | 0.11 | 0.17 | 0.13 |
| CGG(R) | 0.19 | 0.11 | 0.09 | 0.43 | 0.3 | 0.29 | 0.15 | 0.1 |
| CCG(P) | 0.17 | 0.17 | 0.15 | 0.19 | 0.23 | 0.15 | 0.22 | 0.12 |
| GCG(A) | 0.15 | 0.25 | 0.22 | 0.32 | 0.2 | 0.1 | 0.24 | 0.1 |
| GGG(G) | 0.12 | 0.18 | 0.16 | 0.31 | 0.24 | 0.14 | 0.11 | 0.09 |
| UCG(S) | 0.11 | 0.25 | 0.21 | 0.19 | 0.19 | 0.09 | 0.15 | 0.06 |
