# Supplementary material for: Characterization of codon usage pattern in SARS-CoV-2
Source: Virol J. 2020 Sep 14;17:138. doi: 10.1186/s12985-020-01395-x (PMC7487440; doi:10.1186/s12985-020-01395-x)
Supplement: Supplementary file 20 — Additional file 20: Table S3. 100 complete genome of SARS-CoV-2 isolates analyzed in this study. [file 12985_2020_1395_MOESM20_ESM.docx]

**Supplementary Table 3**  100 complete genome of SARS-CoV-2 isolates analyzed in this study.

| **GenBank ACCESSION** | **Isolate** | **Genome Length(nt)** | **Collection_Date** | **Locality** |
| --- | --- | --- | --- | --- |
| LC528232.1 | SARS-CoV-2/Hu/DP/Kng/19-020 | 29902 | 2/10/2020 | Japan |
| LC529905.1 | TKYE6182_2020 | 29903 | 2020-01 | Japan |
| LC534418.1 | SARS-CoV-2/Hu/DP/Kng/19-031 | 29878 | 2/14/2020 | Japan |
| LC534419.1 | SARS-CoV-2/Hu/Kng/19-437 | 29874 | 3/9/2020 | Japan |
| LC547518.1 | hCoV-19/Japan/P1/2020 | 29782 | 3/8/2020 | Japan |
| MN908947.3 | Wuhan-Hu-1 | 29903 | 2019-12 | China |
| MN985325.1 | 2019-nCoV/USA-WA1/2020 | 29882 | 1/19/2020 | USA |
| MN994467.1 | 2019-nCoV/USA-CA1/2020 | 29882 | 1/23/2020 | USA |
| MN994468.1 | 2019-nCoV/USA-CA2/2020 | 29883 | 1/22/2020 | USA |
| MT007544.1 | Australia/VIC01/2020 | 29893 | 1/25/2020 | Australia |
| MT019529.1 | BetaCoV/Wuhan/IPBCAMS-WH-01/2019 | 29899 | 12/23/2019 | China |
| MT019530.1 | BetaCoV/Wuhan/IPBCAMS-WH-02/2019 | 29889 | 12/30/2019 | China |
| MT019531.1 | BetaCoV/Wuhan/IPBCAMS-WH-03/2019 | 29899 | 12/30/2019 | China |
| MT019532.1 | BetaCoV/Wuhan/IPBCAMS-WH-04/2019 | 29890 | 12/30/2019 | China |
| MT019533.1 | BetaCoV/Wuhan/IPBCAMS-WH-05/2020 | 29883 | 1/1/2020 | China |
| MT020880.1 | 2019-nCoV/USA-WA1-A12/2020 | 29882 | 1/25/2020 | USA |
| MT020881.1 | 2019-nCoV/USA-WA1-F6/2020 | 29882 | 1/25/2020 | USA |
| MT027062.1 | 2019-nCoV/USA-CA3/2020 | 29882 | 1/29/2020 | USA |
| MT027063.1 | 2019-nCoV/USA-CA4/2020 | 29882 | 1/29/2020 | USA |
| MT027064.1 | 2019-nCoV/USA-CA5/2020 | 29882 | 1/29/2020 | USA |
| MT039873.1 | HZ-1 | 29833 | 1/20/2020 | China |
| MT039890.1 | SNU01 | 29903 | 2020-01 | South Korea |
| MT044257.1 | 2019-nCoV/USA-IL2/2020 | 29882 | 1/28/2020 | USA |
| MT050493.1 | SARS-CoV-2/human/IND/166/2020 | 29851 | 1/31/2020 | India |
| MT066175.1 | SARS-CoV-2/NTU01/TWN/human/2020 | 29870 | 1/31/2020 | Taiwan |
| MT066176.1 | SARS-CoV-2/NTU02/TWN/human/2020 | 29870 | 2/5/2020 | Taiwan |
| MT072688.1 | SARS-CoV-2/human/NPL/61-TW/2020 | 29811 | 1/13/2020 | Nepal |
| MT093571.1 | SARS-CoV-2/human/SWE/01/2020 | 29886 | 2/7/2020 | Sweden |
| MT106052.1 | 2019-nCoV/USA-CA7/2020 | 29882 | 2/6/2020 | USA |
| MT106053.1 | 2019-nCoV/USA-CA8/2020 | 29882 | 2/10/2020 | USA |
| MT118835.1 | 2019-nCoV/USA-CA9/2020 | 29882 | 2/23/2020 | USA |
| MT123291.2 | SARS-CoV-2/human/CHN/IQTC02/2020 | 29882 | 1/29/2020 | China |
| MT123293.2 | SARS-CoV-2/human/CHN/IQTC03/2020 | 29871 | 1/29/2020 | China |
| MT126808.1 | SARS-CoV-2/human/BRA/SP02/2020 | 29876 | 2/28/2020 | Brazil |
| MT135041.1 | SARS-CoV-2/human/CHN/105/2020 | 29903 | 1/26/2020 | China |
| MT135042.1 | SARS-CoV-2/human/CHN/231/2020 | 29903 | 1/28/2020 | China |
| MT135043.1 | SARS-CoV-2/human/CHN/233/2020 | 29903 | 1/28/2020 | China |
| MT135044.1 | SARS-CoV-2/human/CHN/235/2020 | 29903 | 1/28/2020 | China |
| MT152824.1 | SARS-CoV-2/human/USA/WA2/2020 | 29878 | 2/24/2020 | USA |
| MT159705.1 | 2019-nCoV/USA-CruiseA-7/2020 | 29882 | 2/17/2020 | USA |
| MT159706.1 | 2019-nCoV/USA-CruiseA-8/2020 | 29882 | 2/17/2020 | USA |
| MT159707.1 | 2019-nCoV/USA-CruiseA-10/2020 | 29882 | 2/17/2020 | USA |
| MT159708.1 | 2019-nCoV/USA-CruiseA-11/2020 | 29882 | 2/17/2020 | USA |
| MT159709.1 | 2019-nCoV/USA-CruiseA-12/2020 | 29882 | 2/20/2020 | USA |
| MT159710.1 | 2019-nCoV/USA-CruiseA-9/2020 | 29882 | 2/17/2020 | USA |
| MT159711.1 | 2019-nCoV/USA-CruiseA-13/2020 | 29882 | 2/20/2020 | USA |
| MT159712.1 | 2019-nCoV/USA-CruiseA-14/2020 | 29882 | 2/25/2020 | USA |
| MT159713.1 | 2019-nCoV/USA-CruiseA-15/2020 | 29882 | 2/18/2020 | USA |
| MT159714.1 | 2019-nCoV/USA-CruiseA-16/2020 | 29882 | 2/18/2020 | USA |
| MT159715.1 | 2019-nCoV/USA-CruiseA-17/2020 | 29882 | 2/24/2020 | USA |
| MT159717.1 | 2019-nCoV/USA-CruiseA-1/2020 | 29882 | 2/17/2020 | USA |
| MT159718.1 | 2019-nCoV/USA-CruiseA-2/2020 | 29882 | 2/18/2020 | USA |
| MT159719.1 | 2019-nCoV/USA-CruiseA-3/2020 | 29882 | 2/18/2020 | USA |
| MT159720.1 | 2019-nCoV/USA-CruiseA-4/2020 | 29882 | 2/21/2020 | USA |
| MT159721.1 | 2019-nCoV/USA-CruiseA-5/2020 | 29882 | 2/21/2020 | USA |
| MT159722.1 | 2019-nCoV/USA-CruiseA-6/2020 | 29882 | 2/21/2020 | USA |
| MT184907.1 | 2019-nCoV/USA-CruiseA-19/2020 | 29882 | 2/18/2020 | USA |
| MT184909.1 | 2019-nCoV/USA-CruiseA-22/2020 | 29882 | 2/21/2020 | USA |
| MT184912.1 | 2019-nCoV/USA-CruiseA-25/2020 | 29882 | 2/17/2020 | USA |
| MT192759.1 | SARS-CoV-2/human/TWN/CGMH-CGU-01/2020 | 29862 | 1/25/2020 | Taiwan |
| MT192765.1 | SARS-CoV-2/human/USA/PC00101P/2020 | 29829 | 3/11/2020 | USA |
| MT192772.1 | SARS-CoV-2/human/VNM/nCoV-19-01S/2020 | 29891 | 1/22/2020 | Viet Nam |
| MT192773.1 | SARS-CoV-2/human/VNM/nCoV-19-02S/2020 | 29890 | 1/22/2020 | Viet Nam |
| MT233523.1 | SARS-CoV-2/human/ESP/Valencia8/2020 | 29782 | 3/4/2020 | Spain |
| MT246450.1 | SARS-CoV-2/human/USA/WA-UW193/2020 | 29872 | 3/13/2020 | USA |
| MT246452.1 | SARS-CoV-2/human/USA/WA-UW195/2020 | 29888 | 3/13/2020 | USA |
| MT246464.1 | SARS-CoV-2/human/USA/WA-UW207/2020 | 29818 | 3/13/2020 | USA |
| MT246467.1 | SARS-CoV-2/human/USA/WA-UW210/2020 | 29899 | 3/14/2020 | USA |
| MT246471.1 | SARS-CoV-2/human/USA/WA-UW214/2020 | 29871 | 3/14/2020 | USA |
| MT246476.1 | SARS-CoV-2/human/USA/WA-UW219/2020 | 29863 | 3/13/2020 | USA |
| MT246478.1 | SARS-CoV-2/human/USA/WA-UW221/2020 | 29889 | 3/14/2020 | USA |
| MT246480.1 | SARS-CoV-2/human/USA/WA-UW223/2020 | 29904 | 3/13/2020 | USA |
| MT246488.1 | SARS-CoV-2/human/USA/WA-UW231/2020 | 29842 | 3/14/2020 | USA |
| MT251976.1 | SARS-CoV-2/human/USA/WA-UW240/2020 | 29899 | 3/14/2020 | USA |
| MT253696.1 | SARS-CoV-2/human/CHN/HZ-162/2020 | 29781 | 1/23/2020 | China |
| MT253697.1 | SARS-CoV-2/human/CHN/HZ-178/2020 | 29781 | 1/23/2020 | China |
| MT253698.1 | SARS-CoV-2/human/CHN/HZ-185/2020 | 29781 | 1/23/2020 | China |
| MT253699.1 | SARS-CoV-2/human/CHN/HZ-477/2020 | 29781 | 1/24/2020 | China |
| MT253700.1 | SARS-CoV-2/human/CHN/HZ-481/2020 | 29781 | 1/25/2020 | China |
| MT253701.1 | SARS-CoV-2/human/CHN/HZ-48/2020 | 29781 | 1/21/2020 | China |
| MT253702.1 | SARS-CoV-2/human/CHN/HZ-49/2020 | 29781 | 1/21/2020 | China |
| MT253703.1 | SARS-CoV-2/human/CHN/HZ-551/2020 | 29781 | 1/25/2020 | China |
| MT253704.1 | SARS-CoV-2/human/CHN/HZ-576/2020 | 29781 | 1/25/2020 | China |
| MT253705.1 | SARS-CoV-2/human/CHN/HZ-60/2020 | 29781 | 1/22/2020 | China |
| MT253707.1 | SARS-CoV-2/human/CHN/HZ-638/2020 | 29781 | 1/25/2020 | China |
| MT253708.1 | SARS-CoV-2/human/CHN/HZ-79/2020 | 29781 | 1/21/2020 | China |
| MT253709.1 | SARS-CoV-2/human/CHN/HZ-90/2020 | 29781 | 1/21/2020 | China |
| MT253710.1 | SARS-CoV-2/human/CHN/HZ-91/2020 | 29781 | 1/21/2020 | China |
| MT258378.1 | SARS-CoV-2/human/USA/CZB-RR057-006/2020 | 29892 | 3/18/2020 | USA |
| MT259235.1 | SARS-CoV-2/human/USA/WA-UW243/2020 | 29751 | 3/13/2020 | USA |
| MT259239.1 | SARS-CoV-2/human/USA/WA-UW247/2020 | 29583 | 3/16/2020 | USA |
| MT259240.1 | SARS-CoV-2/human/USA/WA-UW248/2020 | 29544 | 3/16/2020 | USA |
| MT259241.1 | SARS-CoV-2/human/USA/WA-UW249/2020 | 29682 | 3/16/2020 | USA |
| MT259243.1 | SARS-CoV-2/human/USA/WA-UW251/2020 | 29757 | 3/13/2020 | USA |
| MT259246.1 | SARS-CoV-2/human/USA/WA-UW254/2020 | 29842 | 3/13/2020 | USA |
| MT259249.1 | SARS-CoV-2/human/USA/WA-UW257/2020 | 29821 | 3/13/2020 | USA |
| MT259252.1 | SARS-CoV-2/human/USA/WA-UW260/2020 | 29790 | 3/14/2020 | USA |
| MT259253.1 | SARS-CoV-2/human/USA/WA-UW261/2020 | 29813 | 3/16/2020 | USA |
| MT259254.1 | SARS-CoV-2/human/USA/WA-UW262/2020 | 29887 | 3/16/2020 | USA |
| MT259256.1 | SARS-CoV-2/human/USA/WA-UW264/2020 | 29833 | 3/13/2020 | USA |
